# Using photovoice to engage underserved children with neurodevelopmental disorders and their caregivers in health research: a mixed methods systematic review

**DOI:** 10.3389/fresc.2025.1638513

**Published:** 2025-08-15

**Authors:** Miriam Gonzalez, Paul Y. Yoo, Samantha E. Noyek, Brooke MacLeod, Michelle Kee, Michelle Phoenix, Samantha K. Micsinszki, Marion Knutson, Christine J. Neilson, Roberta L. Woodgate

**Affiliations:** ^1^ College of Nursing, Rady Faculty of Health Sciences, University of Manitoba, Winnipeg, MB, Canada; ^2^ Division of Neurosciences and Mental Health, SickKids Research Institute, The Hospital for Sick Children, Toronto, ON, Canada; ^3^ Department of Pediatrics, McMaster University, Hamilton, ON, Canada; ^4^ Department of Educational and Counselling Psychology, Faculty of Education, McGill University, Montreal, QC, Canada; ^5^ School of Rehabilitation Science and CanChild, McMaster University, Hamilton, ON, Canada; ^6^ Patient Advisors Network, Virtual Community of Practice for Patient/Caregiver Partners, Canada; ^7^ Neil John Maclean Health Sciences Library, University of Manitoba, Winnipeg, MB, Canada

**Keywords:** photovoice, neurodevelopmental disorders, children, caregivers, underserved, arts-based method

## Abstract

**Introduction:**

Limited guidance exists for researchers wanting to use photovoice to engage children with neurodevelopmental disorders (NDDs), 0-25 years, and their caregivers in health research. This mixed-methods systematic review synthesized photovoice research with this population with attention to children and caregivers from diverse backgrounds. Diversity of study participants, research areas that have used photovoice with this population, feasibility considerations (adaptations, contextual considerations, practicality), and recommendations provided by study authors were of interest.

**Methods:**

We searched five databases and limited the search to English or French language publications. Eighteen studies met the inclusion criteria. We used a convergent integrated synthesis approach as well as qualitative content analysis to synthesize data from included studies.

**Results:**

The majority of children and caregivers in selected studies were white. Selected studies focused primarily on autism spectrum disorder (*n* = 12) followed by intellectual disability (*n* = 3). Photovoice has been used across six research areas relevant to individual, interpersonal, and organizational level influences on an individual's life. Authors of selected studies faced various contextual considerations (e.g., requiring flexibility) and made adaptations (e.g., using smiley/sad faces to monitor assent) to facilitate research participation. Authors reported photovoice as valuable and useful and provided implementation recommendations (e.g., work one-on-one with participants) and future research directions (e.g., using photovoice with nonverbal children) to advance the use of this methodology.

**Discussion:**

Our findings support using photovoice to explore the lived experience of this population, provide guidance to health and rehabilitation researchers seeking inclusive, person-centred approaches to engaging participants in research, and have direct implications for practice.

**Systematic Review Registration:**

https://osf.io/3xsak/

## Introduction

Neurodevelopmental disorders (NDDs) are a group of conditions that begin during the developmental period, are attributed to impairment of the brain and/or neuromuscular system and are associated with functional limitations (1–3). NDDs encompass a broad group of diagnoses including communication disorders, autism spectrum disorder, specific learning disorder, intellectual disability, attention deficit/hyperactivity disorder, and motor disorders (4). Children with NDDs and their caregivers experience health and social disparities that put them at risk of high morbidity, premature mortality, and suboptimal quality of life (5–7). These health and social disparities may be heightened for diverse families or those with multiple and intersecting identities (8–10). Children with NDDs and their caregivers, including diverse families, are thus an underserved group that face disparate health care access and outcomes due to health characteristics (e.g., disorders/disability), sociodemographic status (e.g., ethnicity), racial bias, or other forms of discrimination (4, 11–14).

Engaging underserved groups in research is necessary for advancing equity in research and developing inclusive, equitable, and person-centred health and rehabilitation practices. However, traditional research methods fall short in capturing the lived experiences of these populations. Participatory research methods that are flexible, accessible, and healing-centred offer a promising approach to bridge this gap (15, 16). These methods not only offer creative ways of expressing lived experience but have well-documented therapeutic benefits (17–19, 20–23). Photovoice is a visual, arts-based research methodology developed by Wang and Burris (24) that involves participants using cameras to document and share their perspectives and experiences with photographs. Grounded in participatory inquiry, photovoice is reflective of its ontological and epistemological underpinnings: participants as experts of their experiences and co-creating knowledge through active and meaningful participation (24, 25). In addition to facilitating reflection, documentation, and sharing of experiences, other goals of photovoice include promoting critical dialogue and knowledge about a given topic and reaching policymakers (24). Wang and Burris (25) proposed nine prescriptive implementation steps: (1) Identify and select the target audience of research outcomes (i.e., policymakers or community leaders), (2) recruit research participants, (3) provide potential participants with an overview of the project's objectives and protocols during a group introductory session, (4) obtain participant consent, (5) develop the theme(s) or focus of the photographs, (6) provide cameras and relevant training to participants, (7) provide time to take photographs, (8) facilitate a discussion with participants regarding their photographs, and (9) plan how to present photographs and research outcomes with the target audience (25). During step 8, the SHOWeD method may be used. This method is a questioning technique developed by Wang (25) and involves asking five structured questions [see Wang (25) for details]. The SHOWeD method aims to guide participants in a critical analysis of their photographs and the meaning behind them.

Research using the photovoice methodology has grown in the last three decades (26). The methodology has been applied with the general population (27, 28) and diverse and underserved subgroups (21, 22, 29, 30–38) including individuals with disabilities (39–41). When it comes to NDDs, three reviews have explored the use of photovoice with individuals with specific NDDs. A scoping review (42) and a meta-synthesis of qualitative research (43) explored the use of photovoice with individuals with autism spectrum disorder (ASD). The meta-synthesis included individuals 26 years of age or younger (43) while the scoping review focused on individuals of all ages (42). A systematic review (44) also examined photovoice research with individuals with intellectual disorders across all age groups. All three reviews included studies that used photovoice or photo-elicitation (search strategy included photovoice and photo-elicitation). No systematic review has synthesized the literature on the use of photovoice with children and youth across NDDs. There is also a dearth of knowledge about the use of photovoice with diverse NDD families. But is it feasible to use photovoice with NDD families?. Feasibility research has been conceptualized in various ways but generally refers to research that examines the process and mechanisms that contribute to successful implementation of a given methodology (45–48), study or intervention (49–51). Although various dimensions of feasibility have been proposed (49–52), the dimensions of practicality and adaptation were of key interest. In the context of this review, practicality refers to whether authors reported that photovoice can be used given contextual constraints (e.g., time, available resources) while adaptation refers to the modifications authors reported making to accommodate the context (49, 50). Thus, feasibility considerations of interest included contextual considerations, adaptations, and practicality as reported by authors. To the best of our knowledge, no systematic review has been published with this focus. The overall objective of this review was to synthesize and appraise the best available evidence on the use of photovoice to engage children with neurodevelopmental disorders (0–25 years) and their caregivers in health research with attention to children and caregivers from diverse backgrounds. Specific objectives were:

(1) To synthesize the research areas that have used photovoice with underserved children with NDDs and their caregivers and to examine the extent and nature of participant diversity across these studies. Specific questions were: Which areas of health research have used photovoice when the population under study are children with NDDs and their caregivers? What are the demographics of children with NDD and their caregivers in the studies?
(2) To assess feasibility considerations (contextual considerations, adaptations, practicality) when using photovoice with this population. Specific questions were: Which contextual factors have been reported to influence the feasibility of photovoice with this population and how? How have researchers adapted photovoice to meet the needs of this population? How do authors evaluate the practicality of using photovoice in these contexts?
(3) To derive evidence-informed recommendations for future photovoice projects and research. Specific questions were: Which recommendations have been proposed by study authors that may be helpful for future projects using photovoice with this population? Which future research priorities have been identified to advance the use of photovoice with this population?

## Methods

The reporting of this review was guided by the Preferred Reporting Items for Systematic Reviews and Meta-Analyses (PRISMA) guidelines (See Supplementary Material Table 1) (53). We used a convergent integrated mixed methods systematic review design and a convergent integrated synthesis approach, in accordance with JBI methodology for Mixed Methods Systematic Reviews (54, 55). We also registered a protocol for the review with Open Science Framework (Registration date: June 2024: https://osf.io/3xsak/).

### Stakeholder engagement

We worked with two parents with lived and living experience of neurodevelopmental disorders on this review to ensure our work and findings were relevant to NDD families. Both parents were recruited through team members and have experience partnering with researchers on projects. One parent provided feedback on the protocol, search strategy, screening forms, data extraction forms, and on the descriptive presentation of the data (e.g., Table 1). The other parent worked with us on an infographic for families based on our findings.

**Table 1:** Study and sample characteristics of the 18 included studies.

Sample characteristics	Number of studies	Percentage (%)
Article type		
Qualitative	16	88.9%
Quantitative	–	–
Mixed methods	2	11.1%
Country		
USA	8	44.4%
Canada	1	5.6%
Australia	4	22.2%
UK	3	16.7%
Vietnam	1	5.6%
Ireland	1	5.6%
Recruitment setting		
Community organization (e.g., ASD centre)	8	44.4%
Education setting (e.g., primary school)	4	22.2%
Clinic	2	11.1%
Various (e.g., networks, schools, online platforms)	2	11.1%
Not reported	2	11.1%
Population (sample size)		
Youth	10	55.6%
Children and youth	2	11.1%
Children	1	5.6%
Caregivers only	2	11.1%
Youth and caregivers	2	11.1%
Young adults	1	5.6%
Population subgroup^a^		
White	7	38.9%
Indigenous	1	5.6%
Living in Vietnam	1	5.6%
Rural	2	11.1%
Mixed (rural & urban)	1	5.6%
Caregivers in two-parent households	1	5.6%
Father	–	–
Racialized/visible minority	–	–
Immigrant	–	–
Low-income	–	–
Parent/Caregiver has a disability	–	–
Parent/Caregiver is a language minority	–	–
Parent/Caregiver identifies as 2SLGBTQIA+	–	–
Type of NDD (primary diagnosis)		
ASD	12	66.7%
ID	3	16.7%
Cerebral palsy	1	5.6%
FASD	1	5.6%
Multiple NDDs	1	5.6%
Child age		
Reported child age Mean: 15 years Range: 5–23 years	14	77.8%
Reported range but no data to calculate average Mean: – Range: 7–24 years	4	22.2%
Child gender		
Majority male	13	72.2%
All male	3	16.7%
Majority female	1	5.6%
Not reported	1	5.6%
Child verbal ability		
Verbal	12	66.7%
Verbal ability varied (e.g., can answer questions with prompts; can have limited conversations for a short time; nonverbal)	5	27.8%
Not reported	1	5.6%
Caregiver age		
38–63 yrs	1	5.6%
Not reported	17	94.4%
Caregiver gender		
All female	3	16.7%
Majority female	2	11.1%
Not reported	13	72.2%

NDD, neurodevelopmental disorder; ASD, autism spectrum disorder; ID, intellectual disability; CP, cerebral palsy; FASD, fetal alcohol spectrum disorder.

^a^ Population Subgroup: we report on 13 of the 18 selected studies as three studies only reported the age and gender of the youth and no information regarding population subgroup was reported in two studies.

### Inclusion and exclusion criteria

Studies were eligible for inclusion if they involved children (0–25 years of age) with neurodevelopmental disorders (NDDs) or their caregivers of any age, including underserved groups such as fathers, immigrants, refugees, low-income, Indigenous, or racialized families, caregivers with disabilities, language minorities, those in rural/remote communities, or those identifying as 2SLGBTQIA + (Population). Eligible studies used photovoice methodology as defined by Wang and Burris (24) to engage these participants in research (Intervention). We included peer-reviewed qualitative, quantitative, mixed methods, or methods-focused studies published in English or French between 1994 (the date of the first photovoice study (25) and January 2025, with no geographical restrictions (Comparator not applicable). Outcomes of interest included the health research domains in which photovoice was applied, the demographics and diversity of children with NDDs and their caregivers, feasibility considerations (contextual factors, adaptations, practicality), and recommendations or future research directions (Outcomes).

Studies were excluded if they: (1) focused on adults (26 years of age or older) living with a NDD, or (2) focused on parents or caregivers of adults (26 years of age or older) living with a NDD, or (3) focused on clinicians/staff, or (4) were case reports, editorials, conference proceedings, abstracts, reviews, protocols, books, book chapters, commentaries, theses/dissertations, or research briefs. As per the DSM-5 definition of neurodevelopmental disorders (1), NDDs of interest were: intellectual disabilities, communication disorders, autism spectrum disorder, attention deficit hyperactivity disorder, specified learning disorders, motor disorders, Tic Disorders, and other neurodevelopmental disorders (other specified neurodevelopmental disorder, unspecified neurodevelopmental disorder). Our definition of children was informed by the Lancet Commission on Adolescent Health and Well-being and included those aged 0–25 years (56). Caregivers referred to unpaid adult family members (e.g., parents) caring for the person living with a NDD (57).

### Search strategy

The search strategy was developed by a professional librarian (CJN) in Medline, and peer reviewed by a second librarian (RC) using the PRESS checklist as a template (58). We searched five bibliographic databases to identify potentially relevant publications for this review: Ovid MEDLINE(R) and Epub Ahead of Print, In-Process, In-Data-Review & Other Non-Indexed Citations and Daily; Ovid Embase**;** Ovid APA PsycInfo; CINAHL with Full Text (EBSCOhost); and Academic Search Complete (EBSCOhost). The only database limits used for the search was a limit to English- or French-language publications. The search was last conducted on January 08, 2025. Complete search histories for each database are available online (https://doi.org/10.34990/FK2/7J5JNK).

### Study selection

Search results were imported into Covidence (59) to manage review and selection of articles including rationale for exclusion. Duplicate records were removed by the Covidence software and manually. The title and abstract screening form was first pilot tested. Three reviewers (MG, PYY, SEN) screened three articles, discussed ratings, and the screening form was revised based on discussions. To ensure reliability across reviewers, a calibration exercise was completed where all three reviewers screened 10 articles. We used percent agreement as the measure of interrater reliability as all reviewers had training and experience with the screening process (60). Interrater agreement was set at a threshold of 80%. The calibration exercise for the title and abstract screening phase was completed twice at which point percent agreement was 96% across reviewers. Two dyads of reviewers (MG and PYY, MG and SEN) then independently screened the title and abstract of selected studies for relevance. Disagreements were resolved through discussion and in consultation with the senior author on the team (RLW). The same process was used for full text screening. The same two dyads of reviewers (MG and PYY, MG and SEN) completed the calibration exercise twice and achieved inter-rater agreement of 96%. The same reviewers then screened selected studies using a full text screening form that outlined the inclusion criteria. Two reviewers agreed on articles to be included, excluded, and disagreements were resolved through discussion and in consultation with RLW.

### Quality assessment

Two reviewers (MG and PYY) critically appraised each included study independently. Any disagreements that arose between the reviewers were resolved through discussion or with a third reviewer (RLW). The Mixed Methods Appraisal Tool (MMAT) version 2018 (61) was used for this purpose given this version was specifically developed for systematic reviews that integrate qualitative, quantitative, and mixed methods evidence (62, 63). This tool provides methodological quality criteria for five different study designs including qualitative, quantitative, mixed methods studies. For each category of study design, the MMAT provides a checklist of five criteria with response options of “Yes,” “No,” or “Can’t tell.” For qualitative studies, methodological quality criteria include: fit between research approach and research question, adequacy of qualitative data collection methods, whether findings are derived from the data, whether results interpretation is substantiated by data, and coherence between qualitative data sources, collection, analysis, and interpretation (61). Mixed methods studies are appraised using the appropriate criteria for their qualitative, quantitative, and mixed methods components. In line with the recommendations for using this version of the MMAT (61), there were no scores calculated for overall study quality as using a global score is less informative than evaluating quality based on specific criteria. Strengths of the MMAT tool include ease of use (has limited number of core criteria per study design), focus on methodological quality and trustworthiness, and good content validity (61).

### Data extraction and synthesis

Three reviewers (MG, PYY, SEN) first piloted the data collection form. Reviewers extracted data from two of the selected articles, discussed issues that arose while extracting data, and the form was revised based on discussions. One reviewer (MG) then extracted data from selected studies, a second reviewer (PYY) verified the extracted data, and discrepancies were resolved through discussion and in consultation with the senior author on the team (RLW). The following data were extracted: author, year of publication, location (country), research design, recruitment setting, sample population (e.g., youth, caregiver), population subgroup (e.g., immigrant family), sample size, child age, child gender, child disability, child functioning ability, assistive devices used by child (if any), caregiver age, caregiver gender, study research question, health area photovoice was being used to understand, how photovoice was implemented (adaptations made), challenges encountered, author-reported usefulness, and recommendations made to advance the use of photovoice including future research directions.

Study characteristics were synthesized descriptively and presented in tabular form. We used a convergent integrated synthesis approach to synthesize qualitative and quantitative data. In line with this approach, quantitative data were coded (textual descriptions) separately as were the qualitative data (55). The transformation of quantitative data into codes, allowed for the integration with qualitative findings into a single dataset. The combined dataset was then analyzed using a qualitative content analysis approach (64). We chose this analytic approach as it allowed us to identify categories and findings while also documenting frequency of utterances within each category to support interpretation. Our content analysis approach involved three steps: (1) grouping codes into categories, (2) documenting number of utterances per category and studies reporting adaptations or recommendations respectively, and (3) integrating categories into six main findings: health research areas that have used photovoice and diversity of study participants, contextual considerations when using photovoice with this population, adaptations made when using photovoice with this population, practicality of photovoice as reported by authors, recommendations reported by authors, and future research questions suggested by authors. For the adaptations data, we positioned our results within the photovoice steps recommended by Wang and Burris (24) to facilitate contextualizing our findings. The data were coded by one team member (MG), reviewed for congruence of coding by a second team member (RLW), and discrepancies were resolved by discussion until consensus was reached. To enhance methodological rigour, we used careful line-by-line analysis of the extracted data, prolonged engagement with the data, and ensured that two researchers engaged with the data and a third researcher (PYY) was available to resolve discrepancies.

### Amendments to registered protocol

Two changes from the published protocol were made in response to the nature of the final set of included studies and the data extracted. First, the original protocol proposed exploring facilitators to photovoice use. However, during data extraction, it became evident that many “facilitators” were adaptations, a focus already addressed by another review question. To avoid conceptual overlap and redundancy, we reframed this question to examine “practicality” as a dimension of feasibility (49, 50). This shift allowed us to more meaningfully contextualize implementation-related findings (e.g., adaptations). Second, the published protocol proposed using a descriptive synthesis approach. However, given the inclusion of both qualitative and mixed-methods studies, we adopted a convergent integrated synthesis approach and used qualitative content analysis to analyze the combined dataset (as noted in previous section).

## Results

### Study selection

**Figure 1:**
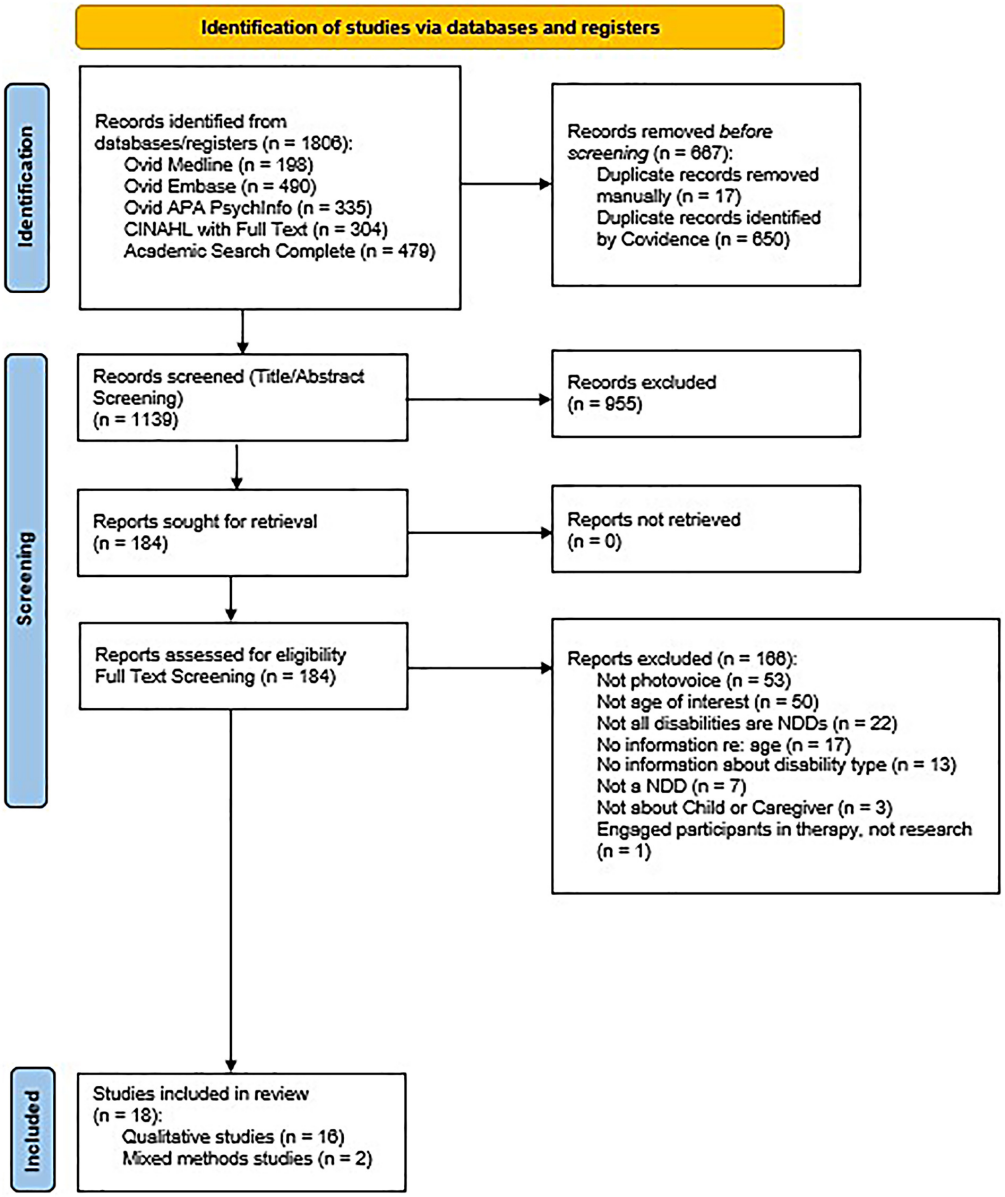
PRISMA 2020 flow diagram for new systematic reviews which included searches of databases and registers only.

The search yielded a total of 1,806 articles, leaving 1,139 articles after duplicates were removed. Based on title and abstract screening, 184 articles were selected for full text screening. Of these, 18 articles met the criteria for inclusion in this review. See Figure 1 for details about the search and selection processes. For information about excluded studies during full text screening, see Supplementary Material Table 2.

### Quality assessment

The MMAT results are shown in Supplementary Material Table 3. Of the 16 qualitative studies, 12 met all MMAT criteria for good quality (65–76), one study lacked quotes and visual data to justify the reported themes (77), and the remaining three studies lacked detailed information about their analytic approach (78–80). The two mixed-methods studies demonstrated overall good quality (81, 82). They satisfied all five MMAT qualitative criteria. When the quantitative components of these studies were assessed, it was determined that their samples were not representative of the target population due to the use of purposive (82) and convenience sampling (81). These studies met four of the five MMAT mixed methods criteria, with neither study providing a rationale for using a mixed methods design. Overall, the studies included in this review were determined to be of good quality. The majority met all relevant criteria with remaining studies falling short in one or two areas, indicating that the research was generally robust.

### Study characteristics

Qualitative Studies (*n* = 16). The qualitative studies (see Table 1) were conducted in Canada, the U.S.A., Australia, Vietnam, the U.K., and the Republic of Ireland. In these studies, participants were recruited through community clinics (66, 67), education settings (65, 68, 73, 75), community organizations (70, 71, 72, 74, 76, 77, 78, 81), or from various soucers (e.g., networks, schools, online platforms) (69, 80). Diagnoses and conditions identified included autism spectrum disorder, intellectual disabilities and/or developmental disabilities, cerebral palsy, and fetal alcohol spectrum disorder. Data collection methods used included semi-structured interviews, photovoice, focus groups, and field observations.

Mixed Methods Studies (*n* = 2). Both mixed-method studies (see Table 1) were conducted in the

USA (81, 82). One study recruited participants from a community organization serving children with autism (81) while the other study's recruitment setting was not reported (82). Cerebral palsy and autism were the disabilities of interest in these studies. Data were collected via photovoice, interviews (semi-structured and in-depth), parent focus groups, standardized scales, accelerometry data, activity logs, and questionnaires developed by researchers. For additional information about the included studies, see Supplementary Material Table 4 and Table 5.

### Findings

We identified six main findings and describe them below.

#### Finding 1: diversity of study participants and research areas that have used photovoice

Sample characteristics are presented in Table 1. The samples of selected studies included children (75), youth (65–68, 70, 76, 80), children and youth (71, 81), caregivers (72, 74), caregivers and youth (79, 82), and caregivers and young adults (77). Over half of the selected studies focused on autism (*n* = 12) (66–68, 70, 71, 73–76, 78–81) followed by intellectual disability (*n* = 3) (65, 67, 77). Similarly, more than half of selected studies included children who were verbal (*n* = 12) (65–71, 75, 76, 79, 81). Children, youth, and young adults in the selected studies ranged in age from 5 to 24 years and the majority were male and white (see Table 1). Caregiver gender and ethnicity was reported in five studies, where caregivers where predominantly female and white (72, 74, 77, 79, 82). The age of the caregivers was only reported in one study, where age ranged from 38 to 63 years (77). In terms of participant diversity, the selected studies focused on: Native American young adults (77), children and youth living in Vietnam (71), white youth (66, 67, 70, 76), white children and youth (75, 81), youth and caregivers living in rural areas (79, 82), caregivers living in both rural and urban areas (72), and caregivers living in two-parent households (74). Overall, the studies reflected limited participant diversity. Most samples comprised white male youth and white female caregivers.

The studies included in this review used photovoice to detail the experience of youth in education settings (65, 80), physical activity participation (81, 82), transitioning into adulthood (66, 67, 70, 76), and to explore their experiences of health and wellness (68, 71, 77) and life experiences such as friendships (69, 71, 79). Photovoice was also used to explore caregivers' experiences of physical activity participation (82), health and wellness (77), and life experiences such as impact of assistance dog ownership (72) and housing adaptations on the family (74). Three studies examined how photovoice can be used to meaningfully engage NDD children in research (73, 75, 78) (see Supplementary Material Table 6).

#### Finding 2: contextual considerations when using photovoice with this population

This finding details key considerations that were needed when implementing the photovoice methodology. These included requiring flexibility and more of everything, taking into consideration the functioning level of the child/youth, unique engagement-related challenges, children/youth fears, difficulty asking for consent and complying with instructions, and challenges related to the analysis and interpretation of photographs. A list of the contextual considerations, number of utterances, and representative quotations can be seen in Supplementary Material Table 7.

##### Requiring flexibility and more of everything

Authors noted the importance of being flexible in terms of how much and how children/youth wished to be involved, adapting the methodology and analysis approach, and being certain that participants fully understood study purpose and procedures. For instance, authors in one study noted: “*Modified forms with pictures were used to communicate with children about the study purpose and informed consent. Even so, it was difficult to always be certain the children fully understood the nature of their participation”* (71)*.* Using photovoice with children and youth with NDDs and their caregivers also required more of everything such as support, resources, time, and patience. As noted by authors in one study: “*Some participants required more support to successfully use their cameras, and others required regular visits to the family home to maintain communication about the project”* (77).

##### Engagement and analysis challenges and child functioning

Authors noted challenges related to engagement and data analysis. These challenges pertained to advocates or gatekeepers working with the team, communication challenges associated with the neurodevelopmental disorder, and limited engagement of the child/youth in contextualizing the photographs (telling stories about their meaning), the analysis process, and in verifying the findings. In one study it was noted: “*None of the students in this study were involved in verifying the findings, as teachers acted as gatekeepers [and] several teachers failed to arrange a meeting for students to verify the findings”* (68)*.* Given the limited contextualization of the photographs and the ambiguities of the visual images, the need for triangulation with other data sources was highlighted by study authors. In one study, it was noted: “*A number of photographs defied initial interpretation. It was only through shared interpretation with the children and those closest to them that these photographs were discovered to carry a wealth of meaning for the children”* (71).

The functioning level of NDD children and youth also influenced their level of contribution.

The comprehension, communication, and cognitive functioning of participants varied. For instance, whereas some children could communicate about their photos, others provided very little information (71). Authors noted the challenge this posed: “*Having a diverse sample of youth with ASD with various functioning levels and communication abilities was challenging as not all participants were able to contribute at the same level”* (66).

##### Fears, asking consent to take photos, and complying with instructions

Other important considerations noted by study authors related to children and youth being afraid of participating in a group setting (e.g., fear of speaking in front of others, of being criticized) and of breaking the rules of photo-taking (see Table 1). As shared in one study: “*Two students chose to use internet images instead of taking their own photographs as they were afraid of unintentionally breaking the rules of taking photographs in school”* (68)*.* In another study, authors noted: *“Youth described apprehension about talking in a group-setting and worried their pictures would be criticized”* (66)*.* Getting the children to ask for consent when taking photographs of others was also challenging as was getting them to comply with photo-taking instructions. As pointed out in one study: “*Our initial instruction for children to photograph particular topics was not workable and instead children freely photographed whatever they wished”* (71).

To summarize, using photovoice with this population required flexibility and support due to challenges related to participant functioning, communication, consent, group participation, and engagement throughout the research process (e.g., verifying findings, photo interpretation).

#### Finding 3: adaptations made when using photovoice with this population

This finding is about the adaptations study authors reported making to the photovoice methodology. We present the adaptations made for each of the nine photovoice steps recommended by Wang & Burris (24) as well as an additional step incorporated in some of the selected studies.

##### Group size, selecting decision-makers, and obtaining informed consent

Wang and Burris (24) recommend photovoice be conducted with groups of seven to ten individuals. In the selected studies, photovoice was conducted individually, in small groups, and in groups of seven or more participants (see Supplementary Material Table 8). Study samples ranged from one to sixteen participants when conducted individually and from two to six participants when conducted in small groups. When conducted in larger groups, study samples ranged from seven to eleven participants: “*Our final sample size included 11 youth with ASD. All youth participants attended an initial group introduction, two group photo-sharing and discussion sessions”* (70).

Wang and Burris (24) propose that participants select a target audience of decision-makers. Ascan be seen in Supplementary Material Table 8, twelve of the selected studies provided no information in this regard and three studies reported no adaptations. In the remaining studies, researchers selected the target audience of decision-makers. As noted in one of these studies: “*As students with ASD may be uncomfortable presenting complex research finding to policy makers, they were assured that their views would be conveyed to the school* via *a report”* (68)*.* Obtaining informed consent from each participant is also recommended by Wang and Burris (24). While in five of the selected studies no information was provided on obtaining informed consent, in four studies, no adaptation was reported. The remaining studies used adapted study information sheets and consent forms. These were described as easy-to-read, accessible, using simple language, and having pictures or guided imagery to help children/youth understand (see Supplementary Material Table 8). In addition, ongoing consent was sought in three studies. To this end, authors reported using traffic light cards or teach-back questions (e.g., please tell me in your own words, what is this study about?). In one of these studies, authors described the process: *“Traffic light cards were used to monitor children's assent to indicate their wishes to continue (green card), pause (orange card), or end (red card) the research encounter”* (75).

Altogether, photovoice was conducted in various group sizes, ranging from individual to large group formats, with most studies aligning with Wang and Burris (24) recommendation of 7–10 participants. While many studies lacked details or adaptations regarding decision-maker selection and informed consent, other studies used researcher-selected audiences and accessible consent materials, includng visual aids and ongoing consent methods.

##### Introducing photovoice, brainstorming photo topics/themes, and distributing cameras

Wang and Burris (24) recommend having a group session to introduce the photovoice methodology and facilitate a discussion about photovoice, cameras, and ethics. As seen in Supplementary Material Table 8, in four of the selected studies, no information was provided in this regard and in three studies, no adaptations were reported. In three studies, this information was presented via powerpoint presentations emails to participants. In the remaining eight studies, this information was presented in a group setting and/or individually and with adaptations: providing a simple introduction about the cameras, giving participants an information handout using simple language and pictures, using an illustrated study information booklet, showing participants samples of photovoice projects via PowerPoint, and reassuring participants that they could use web-based images (see Supplementary Material Table 8). For instance, in one study, authors noted: “*Objectives were accomplished by using PowerPoint slides to explain to the students what their involvement would entail, how to take photographs and transfer them to the computer, and rules on taking photographs in school”* (68).

Wang and Burris (24) recommend photovoice participants brainstorm together about photo topics (themes). As can be seen in Supplementary Material Table 8, in ten of the selected studies, researchers chose the topic for participants while in two studies, no adaptations or no information on photo topic selection was reported. In the six remaining studies, various ways of identifying photo topics were reported: asking participants to take photos of whatever they wished, getting youth and parents to attend separate brainstorming sessions, and using the game called Talk-n-Toss. The excerpt below describes how this game was used: “*Talk-n-toss involved answering questions [about health and wellness] depending on the colour of the beach ball strip their hand landed on and included broad topics related to culture, individual activities, relaxation and social events”* (77).

Distributing cameras to participants is another photovoice step suggested by Wang and Burris (24). In the selected studies, authors asked participants to use their own devices, were open to participants using other creative possibilities (e.g, drawing), or gave participants a disposable or a digital camera. As noted in one study: “*Parents were given a digital camera to complete the Photovoice portion and adolescents were given a tablet with camera functions. Tablets were given to adolescents to improve ease of use”* (82).

In sum, in most studies, photovoice was introducted through group or individual sessions with accessible materias and visual supports. While researchers often selected photo topics, a few studies engaged participants through creative methods like games. Camera distribution varied: participants used personal devices, creative alternatives, or were provided with cameras or tablets.

##### Time for photo-taking and meeting to discuss photos and identify themes

Wang and Burris (24) recommend giving participants a week to take photos. As seen in Supplementary Material Table 8, six of the selected studies reported no information in that regard, five studies reported no adaptation, and the remaining studies reported giving participants options ranging from asking participants to take photos on the spot to ten days and one month: “*The participants were asked if they would like to lead the first author on a tour around their school and playground. They were given an iPad®/tablet to take pictures of whatever they wanted during the tour”* (75).

Wang and Burris (24) also recommend focus groups to discuss photos and identify themes. To discuss photos, study authors used adapted interviews or adapted meetings (see Supplementary Material Table 8). Adaptations made during interviews included using a three-question interview protocol, using open-ended questions, using interview schedules modified for language comprehension, and asking complex questions at the end. As noted in one study: “*The interview schedule had more complex questions at the end of each section. Open-ended questions were selected because they allowed the same questions to be asked using different formats”* (65)*.* Adaptations made during individual or group meetings involved getting children to describe whatever they wanted about their photos, using verbal prompts instead of structured questions, and using PowerPoint presentations where children could type captions about the photos (see Supplementary Material Table 8). As authors in one study described: “*The first author did not use the SHOWeD set of questions since most children found these questions too abstract and hard to answer. Instead, children described whatever they wanted about their photos”* (71).

Wang and Burris (24) also recommend that in a group setting, participants codify or identify themes arising from the photographs. In seven of the selected studies, researchers identified the themes and in two of the studies, participants were not involved in theme identification (see Supplementary Material Table 8). In the remaining studies, researchers identified themes and consulted participants or researchers used adaptations to work with participants to identify themes (see Supplementary Material Table 8). As noted in one study: “*After preliminary analyses, the researcher shared tentative conclusions with the participants in the form of a summary. The researcher provided a brief description of the individual themes, and the participants were asked if they could identify themselves”* (65).

Overall, time given for photo-taking varied: some studies offered on the-spot opportunities while others allowed up to a month. Photo discussions were often adapted for accessibility using simplified interview formats, verbal prompts, or visual aids. In most cases, researchers led the theme identification process, consulting participants or using adaptations to work with participants in identifying themes.

##### Planning a format to share photos and seeking participant feedback

Wang and Burris (24) suggest that both researchers and participants choose the best medium to present the photographs. As can be seen in Supplementary Material 8, in seven of the selected studies, no information was provided in this regard and in ten studies, it was not specified who chose the medium. Only in one study, participants and researchers chose the medium: “*The dissemination process was constructed between the participants and first author. This entailed online meetings to decide if and how to share their photos”* (69)*.* Finally, in eight of the selected studies for this review, study authors reported an additional step: seeking participant feedback regarding their experience with research activities. As can be seen in Supplementary Material Table 8, this was done by conducting an individual interview at the end of the project, filling out a feedback form, or discussing reflection questions with the researcher. In one study, it was noted: “*After the public exhibit, each participant was individually interviewed to explore his or her experiences related to the project. Individual interviews with participants lasted between 45 and 60 min”* (70).

#### Finding 4: practicality of photovoice as reported by authors

This finding refers to study authors reporting that photovoice can be used with children (25 ≤ yrs) living with a NDD and their caregivers despite contextual constraints and adaptations required. In two of the selected studies, no information was provided in this regard. In all remaining studies, authors reported photovoice can be used to help NDD children/youth and their caregivers to express and describe their experiences, to empower them to share their stories, and to engage them meaningfully in research (see Supplementary Material Table 9). In one study authors noted: “*Photovoice proved a useful means of allowing children with ASD and their parents an opportunity to describe their lived experience and engage meaningfully with research”* (71)*.* In another study, authors reflected: “*We demonstrated the utility of a new methodology, Photovoice, that not only assists in the collection of data from an underrepresented population with known communication issues but also serves as a tool to empower youth”* (66).

#### Finding 5: recommendations provided by authors to advance the use of photovoice

This finding details the recommendations provided by study authors for using photovoice in research, in practice settings, and to facilitate meaningful engagement (categories and recommendations are outlined in Supplementary Material Table 10). Although all recommendations provided require further systematic examination, some of the recommendations (implementing photovoice in research) are based on the evidence generated by the selected studies. The remaining recommendations have not been systematically examined and require testing.

##### Implementing photovoice in research

Recommendations for implementing the photovoice methodology in research included: using point-and-shoot cameras with automatic flash to make it easier for participants to take photos, having designated times to meet and share photos, working one-on-one with participants, and working with organization advocates who can work closely with participants. As some authors noted: **“***The use of point-and-shoot cameras with automatic flash may have helped study participants as several youth described feeling nervous about using the cameras”* (66)*.* Other recommendations provided included using one-on-one interviews to ask participants about the photos, avoiding making assumptions about the photographs taken, and using triangulation to assist with photo interpretation. As reported in one study: “*The case studies we describe in this paper demonstrate the need for triangulation with observations and interviews with parents and others in the interpretation of the photographs”* (71).

##### Facilitating meaningful engagement and use in practice settings

Recommendations provided by study author to facilitate the meaningful engagement of children and youth with NDDs and their caregivers in research included using a diversity of data collection approaches, adapting data collection approaches as needed, attending to power dynamics, communicating respect, and recognizing expertise. In one study, authors highlighted: “*It is important to develop a diversity of approaches so that autistic young people can share their ideas, experiences and views in ways that suit their communicative preferences and interests”* (75)*.* Co-creating methods and research foci and providing ownership (control and power) of research materials and processes were also seen as crucial. As noted in another study: “*Participatory, inclusive methods which build in choice and flexibility empower participants and reposition power and control throughout the research process”* (73).

Recommendations for using photovoice in practice settings included helping NDD children and youth express their experiences, perspectives, and needs during clinical encounters as well as in treatment-based interventions: “*Social workers can explore the usefulness of photography and creative art forms as tools to assist youth with ASD in voicing their challenges and solutions”* (70)*.* Authors also saw potential in using photovoice to facilitate engagement in therapy, community activities, and program or policy development. As noted in another study: “*Engaging young adults through photos could allow them, regardless of functioning level, to participate in group or community activities”* (76).

#### Finding 6: future research questions suggested by authors

This finding refers to future research questions suggested by study authors. Suggestions provided pertained to advancing the use of photovoice as well as building and advancing the research base on families affected by a NDD (see Supplementary Material Table 11).

##### Advancing the use of photovoice

To advance the use of photovoice, authors suggested exploring using this methodology with children, youth, and young adults with different impairments. Authors emphasized the need for future research with nonverbal youth, those with significant communication barriers, as well as youth with more severe intellectual or cognitive impairments. In one study, authors noted: “*Future studies should examine how Photovoice can assist nonverbal youth in expressing themselves as well as those with more severe intellectual limitations”* (66)*.* Examining the usefulness of photovoice with distinct segments of the autism spectrum disorder continuum was also seen as important: “*Photovoice may be a particularly helpful method with certain segments of the continuum. Research on distinct segments and ages within the ASD population is warranted”* (76).

##### Building on and advancing the NDD families research base

Study authors suggested examining the experiences of NDD children, youth, and young adults in diverse settings and from diverse backgrounds (see Supplementary Material Table 11). Diverse settings included at the individual and family level. For instance, in one study, it was noted: “*Although familial roles are critical to peer relationships at early ages, less is known about familial roles in adolescent friendship development”* (79)*.* Suggestions for future research at the community level were also reported and included settings such as education (e.g., school), the physical and community environment (e.g., infrastructure), and rural environments. As shared by some authors: “*A more detailed investigation of the characteristics of the physical and community environment that may influence moderate to vigorous physical activity of children with autism spectrum disorder is needed”* (81).

Exploring the experiences of NDD children, youth, and young adults from diverse backgrounds was also highlighted as important. Specific groups highlighted by study authors included those who identify as Indigenous (e.g., Native Americans), racialized or members of an ethnic minority group, gender diverse individuals, and those who live in rural communities. As noted in one study: “*Further study is needed to understand the lives of children with ASD from less privileged backgrounds and in rural communities”* (71)*.* Finally, giving research attention to the needs of individuals with ASD and their families and engaging these families in research and program development were also noted as important for building the knowledge base. As shared by authors in one study: “*Incorporating more opportunities for families, children and adults with CP [cerebral palsy] and other mobility impairments to develop specific programing is crucial to ensure accessibility of programs, equipment, and public spaces”* (82).

## Discussion

This is the first mixed methods systematic review to synthesize the literature on the use of photovoice with children (0–25 years) with neurodevelopmental disorders (NDDs) and their caregivers with attention to examining how the methodology has been used with children and caregivers from diverse backgrounds. Our focus on all NDDs as defined by the Diagnostic and Statistical Manual of Mental Illnesses (DSM-5) and attention to the feasibility of using photovoice with this population (contextual considerations, adaptations, and practicality of photovoice as reported by authors) was also novel. Our review and analysis of 18 studies from the international literature contributes to the knowledge base in this area in critical ways. First, our findings shed light on the diversity of participants in these studies and the need for research with children and caregivers from diverse backgrounds. Second, the findings provide information on feasibility considerations to guide researchers wanting to use this methodology with this population. Finally, through this work, we highlight recommendations provided by study authors (e.g., future research questions, using photovoice with this population) to advance the use of this methodology with NDD children and their caregivers.

### Research areas and diversity of study participants

The selected studies clearly described using photovoice across six research areas. One research area was methodological and reflected studies whose purpose was to describe how photovoice had been used to meaningfully engage our population of interest. The remaining five research areas explored the experiences of children with NDDs or their caregivers in life situations (83): in education settings (65, 80), physical activity participation (81), social life (72, 79), transitioning into adulthood (70), and experiences of health and wellness (68). These research areas focus on individual (e.g., perspectives), interpersonal (e.g., friendship), and organizational (e.g., school) contexts that influence an individual (84, 85). Lacking is photovoice research with NDD families exploring community-level (e.g., design, spaces) and public policy level (e.g., federal, provincial) influences on their lives.

With regards to diversity of study participants, there was limited diversity of participants in the included studies. The majority of the youth were white and male. Where caregiver gender and ethnicity were reported, caregivers were predominantly white and female. Further, no studies were identified that focused on immigrant, refugee, low-income, or racialized families, or caregivers who identify as having a disability, being a language minority, or being 2SLGBTQIA + . The lack of diversity in health research is well-documented (86–89). Our finding regarding the sex and ethnicity of study participants is also consistent with research that indicates that some NDDs (e.g., autism) predominantly affect males (90) and that females are more likely than males to be caregivers (57). Nevertheless, the lack of diverse representation of study participants found in our review highlights the need for research to better understand how photovoice can be adapted to engage these subgroups of the population and make research participation more accessible.

In terms of neurodevelopmental disorders represented in the included studies, the majority of the studies were conducted with children with autism spectrum disorder (ASD) and/or intellectual disabilities (ID). This finding is consistent with the focus of recent reviews that have explored the use of photovoice with people (any age) with ID (44) or individuals (26 years of age or younger) with ASD (43). The remaining studies included in this review focused on cerebral palsy (*n* = 1), fetal alcohol spectrum disorder (*n* = 1), and in one study, children had comorbid NDDs. This highlights the scarcity of and need for photovoice research with other NDDs.

### Feasibility considerations: practicality, contextual considerations, and adaptations

In this review we were interested in examining author-reported practicality of photovoice-that is, whether study authors reported that photovoice can be used with children with NDDs and their caregivers despite contextual constraints (e.g., available resources). In all but two of the included studies (where no information was provided in this regard), authors highlighted that photovoice can be used and described the methodology as valuable in helping participants express their experiences, facilitating meaningful engagement, and promoting empowerment. This finding is consistent with the literature on the feasibility of photovoice for adults with disabilities (47, 91) and other underserved groups (38, 92–94). Further, in eight of the included studies, study participants were asked for feedback about their experience with research activities including taking photos. This feedback was positive (e.g., participants enjoyed the photovoice process) and supports using photovoice to engage this population in research. Synthesizing the literature on NDD families' experiences of using photovoice was beyond the scope of this review as was synthesizing results of studies that use standardized instruments to measure practicality. Research in these two areas would strengthen the validity of our results.

Crucial contextual considerations need to be taken into account when implementing photovoice with children (0–25 years) with NDD and their caregivers. Consistent with our findings, previous literature highlights the challenges of implementing photovoice due to participants' physical and cognitive functioning which leads to difficulty taking photos and talking about the photos (95, 96). However, our findings underscore the need for flexibility, patience, and time to mitigate these difficulties. Another contextual consideration we identified is the provision of additional support required in the form of modification and human assistance in the use of equipment or ascertaining meaning from the photos, consistent with previous literature (91, 97, 98). Fear of criticism when sharing photos in a small group setting or of breaking the rules of photo-taking was another contextual consideration identified. Fear of public scrutiny when working in groups or when sharing photos has been previously documented (91, 97). The contextual considerations we have identified in this review highlight the specific needs of children and youth with NDDs (0–25 years) that may be different from other age groups and conditions.

The adaptations documented in this review reflect flexibility, creativity, and the importance of using a strength-based approach when using photovoice with NDD families. For instance, to express their experiences, participants were asked to take photos using disposable cameras, their own devices, or to choose from a wide range of creative possibilities (e.g., web images, magazine images, drawings). Time provided to take or compile photos was also flexible. The adaptations made in the included studies point to using the nine steps proposed by Wang and Burris (24) as a flexible guide. This is consistent with research that highlights the importance of using photovoice flexibly to meet the needs of project participants (39, 99, 100). A notable finding regarding adaptations is that when discussing photos with participants, only two of the included studies used the SHOWeD method. To discuss photos, study authors adapted interviews or meetings and used creative methods to ask participants about the photos (e.g., used simple verbal prompts, child could describe what they wanted about the photo). This finding corroborates previous work that has highlighted the need for using methods that are creative and person-centred to meet the communication preferences and needs of each child (101–106).

### Recommendations for future photovoice projects

Beyond highlighting the importance of exploring how photovoice can be integrated into practice settings, authors of selected studies provided recommendations for future photovoice research projects. These recommendations point to key messages. First, our findings emphasize the importance of building rapport and developing partnerships with participants and advocates to facilitate active photovoice involvement in studies. Various strategies were suggested including working one-on-one with participants and working closely with advocates (e.g., parents, teachers) to involve them in the research and get them to support children/youth throughout the research process. Meaningful connections can harness trusting relationships, and trust is critically important in the involvement of individuals with disabilities in research (107). In addition, facilitating meaningful research engagement through inclusive strategies (e.g., providing choice and ownership of research process, communicating respect) is crucial and requires thoughtful consideration. Effective, inclusive strategies to this end are needed and necessitate research attention (108–111). Third, interviews can be useful when researchers take the time to find out as much as possible about participants (e.g., needs,), incorporate multiple visits into the study's design, and use specific strategies such as allowing time for repetition and comprehension, rephrasing questions, and pointing to the photos to re-direct participants to the interview topic. This aligns with previous recommendations for interviewing children with disabilities (104, 112, 113) and with literature highlighting interviews as useful for engaging children with disabilities in research (110, 114, 115). Fourth, triangulation and including the child/youth views can prove useful when interpreting photographs. Various strategies were suggested to this end including using multiple data sources and perspectives and ensuring analysis involves a two-step approach (ensuring children are co-creators of meaning during step one and using the co-creation of meaning to inform the analysis conducted by researchers during step two). This recommendation supports the literature advocating for the use of multiple data collection methods and perspectives to assist with the interpretation process (101, 116–120). Strategies for mitigating potential biases introduced by intermediaries should be given careful thought. Photovoice projects should involve children/youth living with neurodevelopmental disorders (NDD) in data interpretation. Finally, these recommendations as well as the feasibility considerations we have documented point to the need to sustain mindful presence when conducting photovoice research with NDD families (121). This means moving through the research with careful forethought, attentive pace, receptive attention, as well as respect, openness, flexibility, empathy, and relational engagement (121). Adopting these qualities can facilitate building rapport, creating a safe and comfortable research space, adapting methods based on the child's strengths and needs, and anticipating and mitigating potential challenges. Being present and attentive to context can also help researchers remain attuned to the various ways children communicate as well as their physical and emotional wellbeing throughout the research process.

### Future research

The results of this review indicate that research is needed about how photovoice can be used with children who have significant verbal or intellectual limitations. Young children, especially those with limited language or cognitive skills, have the potential to be engaged using photovoice given the fewer demands on literacy and language skills than required in other research methods (122). Research in this area can fill gaps that were previously identified in the literature about how young children with disabilities can partner or participate in research (110). Drawing from existing research that has examined the systemic exclusion of young children with disabilities and individuals with significant intellectual and cognitive disabilities may shed light on the ethical and practical considerations that can be applied when using photovoice to increase the inclusion of these populations in research.

Future photovoice research should also focus on the experiences of NDD families from diverse backgrounds and settings. Disability research can run the risk of being conducted in environments that are not typical of every-day life, limiting opportunities for generalizability and meaningful impact. Photovoice can open possibilities for conducting research that is embedded in individuals' environments, empowering participants to capture ideas that are personally meaningful. This can be a vital shift for people who are from geographic, cultural or linguistic communities that are often excluded in research. Future research directions highlighted in this review also point to exploring how photovoice can be used to meaningfully engage families affected by NDDs in health research, service design, and policy. We also believe that it is essential for NDD families to be a part of communities of practice to define research about NDDs and decide how and when to use photovoice (123). Finally, three of the studies included in this review conducted photovoice via virtual platforms. Implementing online photovoice with NDD families is in its nascent stages and has yet to be fully explored.

### Limitations

First, the majority of studies included in this review focused on autism and intellectual disabilities limiting the generalizability of our findings to other NDDs. Second, although the scope of this review was broad and designed to capture photovoice studies with NDD families of diverse backgrounds, studies focusing on diverse families were limited. Third, this review was limited to studies published in English or French. While this may have excluded relevant studies in other languages, the decision was based on the language proficiency of the review team and resource constraints (availability of funds for translation purposes). This may introduce some bias in the results and should be considered when interpreting the findings. Finally, although various dimensions of feasibility have been proposed, we focused on practicality, contextual considerations, and adaptation. Thus, we have synthesized the literature on these three feasibility considerations. Reviews addressing other dimensions of feasibility (e.g., methodology's acceptability, appropriateness) are warranted to provide a more comprehensive picture of the feasibility of photovoice with this population.

## Conclusions

Through this review, we have synthesized feasibility considerations (contextual considerations, adaptations, practicality as reported by authors,) and identified recommendations to guide future photovoice research with NDD children (0–25 years) and their caregivers. We have also outlined future research directions to advance the use of this methodology with this population. Engaging NDD families in research and gaining insight into their first account lived experience is essential for informing policies and practices and advancing health equity. Our findings point to photovoice as a viable method for engaging these families in research. These insights support researchers across health and rehabilitation fields in using photovoice to strengthen the participation of children and youth with NDDs and their caregivers. Sustaining mindful presence when using photovoice with this population can facilitate making methodological decisions rooted in photovoice's ontology and epistemology of meaningful participation.

## Data Availability

The original contributions presented in the study are included in the article/Supplementary Material, further inquiries can be directed to the corresponding author.
