# Supplementary material for: Using photovoice to engage underserved children with neurodevelopmental disorders and their caregivers in health research: a mixed methods systematic review
Source: Front Rehabil Sci. 2025 Aug 15;6:1638513. doi: 10.3389/fresc.2025.1638513 (PMC12394231; doi:10.3389/fresc.2025.1638513)
Supplement: Supplementary file 2 [file Table2.docx]

Supplementary Material Table 2: Articles Excluded During Full Text Screening (n=166)

| **Exclusion Reason** | **Citation** |
| --- | --- |
| **Not photovoice** (n=53) | 1. Lo JWK, Ma JLC. Use of nature in a multi‐family therapy for Chinese families of adolescents with attention deficit hyperactivity disorder: A photo‐elicitation study. Journal of Family Therapy. 2023;45(1):65–80.  2. Pickering DM, Gill P, Reagon C. A kaleidoscope of well-being to authentically represent the voices of children and young people with complex cerebral palsy: a case study series. Disability & Rehabilitation. 2023;1–15.  3. Coussens M, Destoop B, De Baets S, Desoete A, Oostra A, Vanderstraeten G, et al. A Qualitative Photo Elicitation Research Study to elicit the perception of young children with Developmental Disabilities such as ADHD and/or DCD and/or ASD on their participation. PLoS ONE. 2020;15(3):1–20.  4. King MC, Williams EI, Gleeson K. Using photographs to explore self-understanding in adolescent boys with an autism spectrum condition. Journal of Intellectual & Developmental Disability. 2019;44(2):232–43.  5. Zilli C, Parsons S, Kovshoff H. Keys to engagement: A case study exploring the participation of autistic pupils in educational decision‐making at school. British Journal of Educational Psychology. 2020;90(3):770–89.  6. Klein U. No selfies: the social world of autistic male adults as depicted in their everyday photographic practices. Visual Studies. 2023;38(1):17–33.  7. O’Leary S, Moloney M. Understanding the Experiences of Young Children on the Autism Spectrum as They Navigate the Irish Early Years’ Education System: Valuing Voices in Child-Centered Narratives. International Journal of Qualitative Methods. 2020;19:1–11.  8. Correia RA, Seabra-Santos MJ. Family relationships in intellectual disability: Parents’ perceptions as elicited by family photographs. Journal of Intellectual & Developmental Disability. 2023;48(1):1–11.  9. de Groot R, Kaal HL, Stol WP. Studying Problematic Online Behavior of Adolescents With Mild Intellectual Disabilities and Borderline Intellectual Functioning: Methodological and Ethical Considerations for Data Collection. International Journal of Qualitative Methods. 2019;1–10.  10. Hill L. “Some of it I haven’t told anybody else”: Using photo elicitation to explore the experiences of secondary school education from the perspective of young people with a diagnosis of Autistic Spectrum Disorder. Educational & Child Psychology. 2014;31(1):79–89.  11. van Bysterveldt AK, Westerveld MF. Children with Down Syndrome Sharing Past Personal Event Narratives with Their Teacher Aides: A Pilot Study. International Journal of Disability, Development & Education. 2017;64(3):249–69.  12. Krieger B, Piškur B, Schulze C, Beurskens A, Moser A. Environmental pre-requisites and social interchange: the participation experience of adolescents with autism spectrum disorder in Zurich. Disability & Rehabilitation. 2021;43(26):3789–802.  13. Mooney F, Rafique N, Tilly L. Getting involved in the community—What stops us? Findings from an inclusive research project. British Journal of Learning Disabilities. 2019;47(4):241–6.  14. Teachman G, Gibson BE. Integrating Visual Methods With Dialogical Interviews in Research With Youth Who Use Augmentative and Alternative Communication. International Journal of Qualitative Methods. 2018;17(1):1–12.  15. Deakin K, Moore DG, Jahoda A. Children and young people with Down syndrome: Their awareness of Down syndrome and developing self‐perceptions. Journal of Applied Research in Intellectual Disabilities. 2018;31(6):1197–208.  16. Young AF, Chesson RA. Obtaining views on health care from people with learning disabilities and severe mental health problems. British Journal of Learning Disabilities. 2006;34(1):11–9.  17. Paige‐Smith A, Rix J. Researching early intervention and young children’s perspectives - developing and using a “listening to children approach”. British Journal of Special Education. 2011;38(1):28–36.  18. Owen K, Evers C, Hewitt O. Experiences of using a health community team service for people with a learning disability. Learning Disability Practice. 2018;21(5):37–42.  19. Cunningham C, Glenn * S. Self-awareness in Young Adults with Down Syndrome: I. Awareness of Down syndrome and disability. International Journal of Disability, Development & Education. 2004;51(4):335–61.  20. Lamb P, Firbank D, Aldous D. Capturing the world of physical education through the eyes of children with autism spectrum disorders. Sport, Education & Society. 2016;21(5):698–722.  21. Fudge Schormans A. ‘Weightless?’: disrupting relations of power in/through photographic imagery of persons with intellectual disabilities. Disability & Society. 2014;29(5):699–713.  22. Øien I, Fallang B, Østensjø S. Everyday use of assistive technology devices in school settings. Disability & Rehabilitation: Assistive Technology. 2016;11(8):630–5.  23. Orr K, Evans MB, Tamminen KA, Arbour-Nicitopoulos KP. Experiencing the Social Environment of a Canoe Kayak Club: A Case Study of a Special Olympics Program. Case Studies in Sport & Exercise Psychology. 2021;5(1):S1-8.  24. Willis J, Harrison A, Allen JL. Pupils with social, emotional and mental health special needs: Perceptions of how restrictive physical interventions impact their relationships with teaching staff. Teaching & Teacher Education. 2021;97:N.PAG-N.PAG.  25. van Bysterveldt AK, Westerveld MF, Gillon G, Foster‐Cohen S. Personal narrative skills of school-aged children with Down syndrome. International Journal of Language & Communication Disorders. 2012;47(1):95–105.  26. Soto G, Solomon-Rice P, Caputo M. Enhancing the personal narrative skills of elementary school-aged students who use AAC: The effectiveness of personal narrative intervention. Journal of Communication Disorders. 2009;42(1):43–57.  27. Mitchell TK, Bray L, Blake L, Dickinson A, Carter B. “I feel like my house was taken away from me”: Parents’ experiences of having home adaptations for their medically complex, technology‐dependent child. Health & Social Care in the Community. 2022;30(6):e4639–51.  28. King G, Hartman LR, McPherson AC, DeFinney A, Kehl B, Rudzik A, et al. Exploring the after-hours social experiences of youth with disabilities in residential immersive life skills programs: a photo elicitation study. Disability & Rehabilitation. 2022;44(13):3104–12.  29. Hile KA, Santos RM. “Would You Like to Hear a Story?”: Collaborating With Families Using Photo-Elicitation. Journal of Early Intervention. 2022;44(1):40–57.  30. Zazzi H, Faragher R. ‘Visual clutter’ in the classroom: voices of students with Autism Spectrum Disorder. International Journal of Developmental Disabilities. 2018;64(3):212–24.  31. Ibrahim S, Vasalou A, Benton L, Clarke M. A methodological reflection on investigating children’s voice in qualitative research involving children with severe speech and physical impairments. Disability & Society. 2022;37(1):63–88.  32. Vänskä N, Sipari S, Haataja L. What Makes Participation Meaningful? Using Photo-Elicitation to Interview Children with Disabilities. Physical & Occupational Therapy in Pediatrics. 2020;40(6):595–609.  33. Powrie B, Copley J, Turpin M, Ziviani J, Kolehmainen N. The meaning of leisure to children and young people with significant physical disabilities: Implications for optimising participation. British Journal of Occupational Therapy. 2020;83(2):67–77.  34. Cambridge P, Forrester-Jones R. Using individualised communication for interviewing people with intellectual disability: a case study of user-centred research. Journal of Intellectual & Developmental Disability. 2003;28(1):5–23.  35. Westerveld MF, Gillon GT, Moran C. A longitudinal investigation of oral narrative skills in children with mixed reading disability. International Journal of Speech-Language Pathology. 2008;10(3):132–45.  36. Gibson BE, King G, Kushki A, Mistry B, Thompson L, Teachman G, et al. A multi-method approach to studying activity setting participation: integrating standardized questionnaires, qualitative methods and physiological measures. Disability & Rehabilitation. 2014;36(19):1652–60.  37. Yesudian G, Hall M, Owens J, Rodd H, Marshman Z. The oral health experiences of children with learning disabilities in special schools in Sheffield. Journal of Disability & Oral Health. 2012;13(2):45–50.  38. Joy D, Young A, Harvais V, Chesson R. The Use of Photographs as a Means of obtaining Views of Occupational Therapy held by Children with Learning Disabilities: a Pilot Study. British Journal of Occupational Therapy. 1998;61(3):116–20.  39. Lo JW, Ma JL. Generating mutual support in multifamily therapy to promote father involvement and family communication quality of Chinese families of adolescents with Attention Deficit Hyperactivity Disorder: A qualitative study. Family process. 2024 Jun;63(2):967-82.  40. Howe SJ, Hull L, Sedgewick F, Hannon B, McMorris CA. Understanding camouflaging and identity in autistic children and adolescents using photo-elicitation. Research in Autism Spectrum Disorders. 2023 Oct 1;108:102232.  41. Folta SC, Bandini LG, Must A, Pelletier J, Ryan K, Curtin C. Exploring leisure time use and impact on well-being among transition-age autistic youth. Research in Autism Spectrum Disorders. 2022 Aug 1;96:101996.  42. Florindez L.I., Florindez D.C., Price M.E., Florindez F.M., Como D.H., Polido J.C., et al. Exploring eating challenges and food selectivity for latinx children with and without autism spectrum disorder using qualitative visual methodology: Implications for oral health. International Journal of Environmental Research and Public Health. 2021;18(7):3751.  43. Lefebvre M, Chamond J. Phenomenological approach and clinical perspectives on photography with autistic children. Cliniques mediterraneennes: Psychanalyse et Psychopathologie Freudiennes. 2021;103(1):231–44.  44. Pavlopoulou G. A good night’s sleep: Learning about sleep from autistic adolescents’ personal accounts. Frontiers in Psychology. 2021 Apr 7;11:583868.  45. Dias de Faria M, Moreira Casotti L. “Welcome to Holland!” People with Down syndrome as vulnerable consumers. European Journal of Marketing. 2019;53(11):2245–67.  46. Vernay F, Kahina H, Thierry M, Jean-Yves R. Self-paced segmentation of written words on a touchscreen tablet promotes the oral production of nonverbal and minimally verbal children with autism. Journal of Research in Special Educational Needs. 2017;17(4):265–73.  47. Danker J, Strnadova I, Cumming TM. Engaging students with autism spectrum disorder in research through participant-driven photo-elicitation research technique. Australasian Journal of Special Education. 2017;41(1):35–50.  48. Schneider P, Hayward D. Who does what to whom: Introduction of referents in children’s storytelling from pictures. Language, Speech, and Hearing Services in Schools. 2010;41(4):459–73.  49. Owens‐Schill A, Peticca‐Harris A, Elias SRSTA, deGama N. I am because I have to be: Exploring one mother‐worker’s identity of the surrendered self through stories of mothering neurodiverse children. Gender, Work & Organization. 2025;32(1):161–80.  50. Pickering DM, Gill P, Reagon C. A kaleidoscope of well-being to authentically represent the voices of children and young people with complex cerebral palsy: a case study series. Disability & Rehabilitation. 2024;46(7):1339–53.  51. Gulliver K. Listening to young children with disabilities: Experiences of quality in mainstream primary education. British Educational Research Journal. 2024;1.  52. Quartermaine JR, Rose TA, Auld ML, Johnston LM. Reflections on Participation at Home, As Self-Reported by Young People with Cerebral Palsy. Developmental Neurorehabilitation. 2024;27(3/4):69–82.  53. An J, Hodge SR. Exploring the Meaning of Parental Involvement in Physical Education for Students With Developmental Disabilities. Adapted Physical Activity Quarterly. 2013;30(2):147–63. |
| **Not age of interest** (n=50)   - Not all in age group of interest (n=32) | 1. Huggins DW, Copeland SR. Disability and Belonging in an Inclusive Christian Faith Community. Inclusion. 2023;11(4):271–85.  2. Cheah KJ, Riches V, Manokara V. Impact on persons with disabilities and their families when they are contributors to society through volunteerism and employment. Journal of Applied Research in Intellectual Disabilities. 2024;37(2):1–17.  3. Sigstad HMH, Garrels V. A Semi-Structured Approach to Photo Elicitation Methodology for Research Participants With Intellectual Disability. International Journal of Qualitative Methods. 2021;1–6.  4. Benoot T, Dursin W, Verschuere B, Roose R. A visual report on what is of value for people with intellectual disabilities in a Flemish care organisation. Journal of Intellectual & Developmental Disability. 2021;46(4):375–87.  5. Danker J, Strnadová I, Tso M, Loblinzk J, Cumming TM, Martin AJ. “It will open your world up”: The role of mobile technology in promoting social inclusion among adults with intellectual disabilities. British Journal of Learning Disabilities. 2023;51(2):135–47.  6. St. John B, Gray M, Malzacher A, Hladik L, Lurie S, Ausderau K. Using photovoice with people with intellectual disability to illuminate definitions of health and factors influencing participation in health promotion. Journal of Applied Research in Intellectual Disabilities. 2021;34(3):866–76.  7. Weiss JA, Burnham Riosa P, Robinson S, Ryan S, Tint A, Viecili M, et al. Understanding Special Olympics Experiences from the Athlete Perspectives Using Photo-Elicitation: A Qualitative Study. Journal of Applied Research in Intellectual Disabilities. 2017;30(5):936–45.  8. Overmars‐Marx T, Thomése F, Meininger H. Neighbourhood social inclusion from the perspective of people with intellectual disabilities: Relevant themes identified with the use of photovoice. Journal of Applied Research in Intellectual Disabilities. 2019;32(1):82–93.  9. Patka M, Wallin-Ruschman J, bin Nauman H, Ul Hasan T, Ikram S, Sharif JM, et al. Special Olympics Pakistan: using a segregated space to transition to independence in mainstream settings. Disability & Society. 2022;37(10):1591–613.  10. Kattari SK, Beltrán R. “The pain is real”: A [modified] photovoice exploration of disability, chronic pain, and chronic illness (in)visibility. Qualitative Social Work. 2022;21(3):504–22.  11. Garrels V, Sigstad HMH. Motivation for Employment in Norwegian Adults with Mild Intellectual Disability: The Role of Competence, Autonomy, and Relatedness. Scandinavian Journal of Disability Research. 2019;21(1):250–61.  12. Benoot T, Roose R, Dursin W, Verschuere B, McKenzie J. Aspirations of People With Intellectual Disabilities Living in a Care Organization. Journal of Social Work. 2022;22(4):1105–22.  13. Dorozenko KP, Bishop BJ, Roberts LD. Fumblings and faux pas: Reflections on attempting to engage in participatory research with people with an intellectual disability. Journal of Intellectual & Developmental Disability. 2016;41(3):197–208.  14. White S, Kuper H, Itimu-Phiri A, Holm R, Biran A. A Qualitative Study of Barriers to Accessing Water, Sanitation and Hygiene for Disabled People in Malawi. PLoS ONE. 2016;11(5):1–18.  15. Akkerman A, Janssen CGC, Kef S, Meininger HP. Perspectives of Employees with Intellectual Disabilities on Themes Relevant to Their Job Satisfaction. An Explorative Study using Photovoice. Journal of Applied Research in Intellectual Disabilities. 2014;27(6):542–54.  16. Boulton NE, Williams J, Jones RSP. Intellectual disabilities and ACT: feasibility of a photography-based values intervention. Advances in Mental Health & Intellectual Disabilities. 2018;12(1):11–21.  17. O’Brien P, Shevlin M, O’Keefe M, Fitzgerald S, Curtis S, Kenny M. Opening up a whole new world for students with intellectual disabilities within a third level setting. British Journal of Learning Disabilities. 2009;37(4):285–92.  18. Lois Mosquera M, Mandy W, Pavlopoulou G, Dimitriou D. Autistic adults’ personal experiences of navigating a social world prior to and during Covid-19 lockdown in Spain. Research in Developmental Disabilities. 2021;117:N.PAG-N.PAG.  19. Moriña A, Molina VM, Cortés-Vega MD. Voices from Spanish students with disabilities: willpower and effort to survive university. European Journal of Special Needs Education. 2018;33(4):481–94.  20. Dorozenko KP, Roberts LD, Bishop B. The identities and social roles of people with an intellectual disability: challenging dominant cultural worldviews, values and mythologies. Disability & Society. 2015;30(9):1345–64.  21. Ottmann G, Crosbie J. Mixed method approaches in open-ended, qualitative, exploratory research involving people with intellectual disabilities: A comparative methods study. Journal of Intellectual Disabilities. 2013;17(3):182–97.  22. Longhurst P, Aspell J, Todd J, Swami V. “There’s No Separating My View of My Body from My Autism”: A qualitative study of positive body image in autistic individuals. Body Image. 2024 Mar 1;48:101655.  23. Safari M.C., Wass S., Thygesen E. “I got to answer the way I wanted to”: Intellectual disabilities and participation in technology design activities. Scandinavian Journal of Disability Research. 2021;23(1):192–203.  24. Krutt H, Dyer L, Arora A, Rollman J, Jozkowski AC. PhotoVoice is a feasible method of program evaluation at a center serving adults with autism. Evaluation and program planning. 2018 Jun 1;68:74-80.  25. Scheffers F, Moonen X, van Vugt E. Photographic visualization of stories: documenting the experiences of people with intellectual disabilities with guided photovoice. Journal of intellectual disabilities : JOID. 2024;(101229024):17446295241229002.  26. Davidson A, Pfeiffer B. Community Participation Challenges for Young Adults with Autism Spectrum Disorders During COVID-19 A Photovoice Study. Community mental health journal. 2024;60(1):60–71.  27. Hoyle JN, Warren‐Findlow J, Wallace L, Laditka JN, Laditka SB. “It’s Not Like a One‐Way Street”: Using Photovoice to Understand How College Students With Intellectual Disability Experience Interdependence. Journal of Applied Research in Intellectual Disabilities. 2024;37(6):1–11.  28. Cheah KJ, Riches V, Manokara V. Impact on persons with disabilities and their families when they are contributors to society through volunteerism and employment. Journal of Applied Research in Intellectual Disabilities. 2024;37(2):1–17.  29. Paton J., Carrington A., Gentle E., Horsefall D. “I am more than my diagnosis”: Amplifying the voice of consumers in the design and delivery of mental health services. The Australian journal of rural health. 2024;32(6):1140EP – 1149.  30. Elinder LS, Brunosson A, Bergström H, Hagströmer M, Patterson E. Validation of personal digital photography to assess dietary quality among people with intellectual disabilities. Journal of intellectual disability research. 2012 Feb;56(2):221-6.  31. van Nistelrooij I, Niemeijer A. Living in an “ordinary” neighborhood? A care-ethical exploration of the experiences of young adults with mild intellectual disabilities. Disability & society. 2023;38(4):635–58.  32. Scheffers F, van Vugt E, Moonen X. Resilience in the face of adversity: How people with intellectual disabilities deal with challenging times. Journal of Intellectual Disabilities. 2024;28(3):661–82. |
| - Not age of interest (n=16) | 1. Braun AMB, Naami A. Access to Higher Education in Ghana: Examining Experiences through the Lens of Students with Mobility Disabilities. International Journal of Disability, Development & Education. 2021;68(1):95–115.  2. Wos K, Baczała D. Parenting by mothers with intellectual disabilities in Poland: A photovoice study. Journal of Applied Research in Intellectual Disabilities. 2021;34(6):1452–62.  3. St. John BM, Hladik E, Romaniak HC, Ausderau KK. Understanding health disparities for individuals with intellectual disability using photovoice. Scandinavian Journal of Occupational Therapy. 2018;25(5):371–81.  4. Heffron JL, Spassiani NA, Angell AM, Hammel J. Using Photovoice as a participatory method to identify and strategize community participation with people with intellectual and developmental disabilities. Scandinavian Journal of Occupational Therapy. 2018;25(5):382–95.  5. Overmars‐Marx T, Thomése F, Moonen X. Photovoice in research involving people with intellectual disabilities: A guided photovoice approach as an alternative. Journal of Applied Research in Intellectual Disabilities. 2018;31(1):e92–104.  6. Spassiani NA, Meisner BA, Abou Chacra MS, Heller T, Hammel J. What is and isn’t working: Factors involved in sustaining community‐based health and participation initiatives for people ageing with intellectual and developmental disabilities. Journal of Applied Research in Intellectual Disabilities. 2019;32(6):1465–77.  7. van Heumen L, Schippers A. Quality of life for young adults with intellectual disability following individualised support: Individual and family responses. Journal of Intellectual & Developmental Disability. 2016;41(4):299–310.  8. Humphries K, Traci MA, Seekins T. Food on Film: Pilot Test of an Innovative Method for Recording Food Intake of Adults with Intellectual Disabilities Living in the Community. Journal of Applied Research in Intellectual Disabilities. 2008;21(2):168–73.  9. Janine M.  Jurkowski. Photovoice With Vulnerable Populations: Addressing Disparities in Health Promotion Among People With Intellectual Disabilities. Health Promotion Practice. 2007;8(4):358–65.  10. Jurkowski JM, Rivera Y, Hammel J. Health Perceptions of Latinos With Intellectual Disabilities: The Results of a Qualitative Pilot Study. Health Promotion Practice. 2009;10(1):144–55.  11. Savage M.N., Colombo-Dougovito A.M. Capabilities, Opportunities, and Motivation: Exploring Fitness Program Experiences of Adults with Intellectual and Developmental Disabilities. International Journal of Environmental Research and Public Health. 2023;20(10):5771.  12. Nabors L, Sanyaolu O, Adabla S, Ghussin D, Ayers K. Evaluation of the Eat and Exercise to Win Program: Improving healthy behaviors of adults with developmental and intellectual disabilities. Advances in Neurodevelopmental Disorders. 2023;7(1):107–22.  13. Rinaldi J. What survivors see: Creative condemnations of total institutionalization. Emotion, Space and Society. 2021;40(Abbas, J., Voronka, J. (2014). Remembering institutional erasures: the meaning of histories of disability incarceration in Ontario. In L. Ben-Moshe, C. Chapman, A.C. Carey (Eds.), Disability Incarcerated: Imprisonment and Disability in the United Stat).  14. Chinn D, Levitan T, Power A, Brickley K, Ali S. What does “feeling at home” mean for adults with intellectual disabilities living in group homes in England? Journal of Applied Research in Intellectual Disabilities. 2024;37(5):1–14.  15. Malka M, Fine M. The lived experience of parents of children with disabilities as service users: A transformative learning theory perspective. American Journal of Orthopsychiatry. 2024 Sep 19.  16. Smaniotto B., Mauran-Mignorat M., Guenoun T. Desires and representations of desires among persons with intellectual disability. Annales Medico-Psychologiques [Internet]. |
| - Not reporting by age group (n=2) | 1. Patka M, Wallin‐Ruschman J, Al Rahma BA, Zar A, Nauman H, Sharif JM, et al. “We need to share our stories”: the lives of Pakistanis with intellectual disability and their guardians. Journal of Intellectual Disability Research. 2020;64(5):345–56.  2. Povee K, Bishop BJ, Roberts LD. The use of photovoice with people with intellectual disabilities: reflections, challenges and opportunities. Disability & Society. 2014;29(6):893–907. |
| **Not all disabilities are NDDs (n=22)**   - Not all disabilities are NDDs (n=15) | 1.Oulton K, Gibson F, Kenten C, Russell J, Carr L, Hassiotis A, et al. Being a child with intellectual disabilities in hospital: The need for an individualised approach to care. Journal of Applied Research in Intellectual Disabilities.  2023;1.  2. Hepperlen RA, Rabaey P, Ament‐Lemke A, Manley H. Caring for a child with a disability in a Zambian community: A study using photo‐elicitation. Child: Care, Health & Development. 2021;47(4):422–34.  3. Simpson J, Yantzi N, Shute T, Watson S. ‘Even if it’s flawed it’s still beautiful’: life lessons learned by adolescents with neurological conditions at summer camp. Disability & Society. 2021;1–24.  4. Lindsay S, Morales E, Yantzi N, Vincent C, Howell L, Edwards G. The experiences of participating in winter among youths with a physical disability compared with their typically developing peers. Child: Care, Health & Development. 2015;41(6):980–8.  5. Carnahan CR. Photovoice: Engaging Children With Autism and Their Teachers. Teaching Exceptional Children. 2006;39(2):44–50.  6. Dyches TT, Cichella E, Olsen SF, Mandleco B, Koegel LK. Snapshots of Life: Perspectives of School-Aged Individuals with Developmental Disabilities. Research & Practice for Persons with Severe Disabilities. 2004;29(3):172–82.  7. Lassetter JH, Mandleco BL, Roper SO. Family Photographs: Expressions of Parents Raising Children With Disabilities. Qualitative Health Research. 2007;17(4):456–67.  8. Merrick R, Roulstone S. Children’s views of communication and speech-language pathology. International Journal of Speech-Language Pathology. 2011;13(4):281–90.  9. den Besten J, Cornielje MT, Cornielje H, Botwey DN. Supporting Parents in Caring for Children with Disability in Ghana. Disability, CBR & Inclusive Development. 2016;27(3):87–101.  10. Nguyen XT, Mitchell C, de Lange N, Fritsch K. Engaging girls with disabilities in Vietnam: making their voices count. Disability & Society. 2015;30(5):773–87.  11. Woodgate RL, Edwards M, Ripat JD, Borton B, Rempel G. Intense parenting: a qualitative study detailing the experiences of parenting children with complex care needs. BMC Pediatrics. 2015;15:1–15.  12. Harding J, Harding K, Jamieson P, Mullally M, Politi C, Wong-Sing E, et al. Children with disabilities’ perceptions of activity participation and environments: a pilot study. Canadian Journal of Occupational Therapy. 2009;76(3):133–44.  13. Bray L. The use of photography in research. Paediatric Nursing. 2007;19(8):10–10.  14. Woodgate R.L., Isaak C., Kipling A., Kirk S., Keilty K. Respite care: qualitative arts-based findings on the perspectives and experiences of families of children and youth with special healthcare needs residing in Manitoba, Canada. BMJ Open. 2023;13(6):e073391.  15. Robillard C., Cormier I., Dhoot T., Elbaz S., Harrison P., Scazzosi E. The Real Me: Insight into youths’ abilities to transition to adulthood through digital images. Developmental Medicine and Child Neurology. 2022;64(SUPPL 3):35. |
| - Not all have a NDD (n=6) | 1. Bonati ML, Andriana E. Amplifying children’s voices within photovoice: Emerging inclusive education practices in Indonesia. British Journal of Learning Disabilities. 2021;49(4):409–23.  2. Andriana E, Evans D. Voices of students with intellectual disabilities: Experiences of transition in “inclusive schools” in Indonesia. British Journal of Learning Disabilities. 2021;49(3):316–28.  3. Aitken SC, Wingate J. A preliminary study of the self-directed photography of middle-class, homeless, and Mobility-Impaired Children*. Professional Geographer. 1993;45(1):65.  4. Kristensen KL, Mørck L. ADHD medication and social self-understanding. European Journal of Psychology of Education - EJPE (Springer Science & Business Media BV). 2016;31(1):43–59.  5. Heerings M, van de Bovenkamp H, Cardol M, Bal R. Burden of support: a counter narrative of service users’ experiences with community housing services. Disability & Society. 2024;39(3):743–66.  6. Simpson J, Yantzi N, Shute T, Watson S. “Even if it’s flawed it’s still beautiful”: life lessons learned by adolescents with neurological conditions at summer camp. Disability & Society. 2024;39(1):16–39. |
| - Findings not reported by disability type (n=1) | 1. Morales E, Lindsay S, Edwards G, Howell L, Vincent C, Yantzi N, et al. Addressing challenges for youths with mobility devices in winter conditions. Disability & Rehabilitation. 2018;40(1):21–7. |
| **No information re: age** (n=17) | 1. Heerings M, van de Bovenkamp H, Cardol M, Bal R. Ask us! Adjusting experience‐based codesign to be responsive to people with intellectual disabilities, serious mental illness or older persons receiving support with independent living. Health Expectations. 2022;25(5):2246–54.  2. Krisson E, Qureshi M, Head A. Adapting photovoice to explore identity expression amongst people with intellectual disabilities who have limited or no verbal communication. British Journal of Learning Disabilities. 2022;50(1):41–51.  3. Ross T, Buliung R, Murphy A, Howard A. A visual ethnographic pilot study of school travel for families living with childhood disability. Children’s Geographies. 2020;18(3):283–97.  4. Lam GYH, Holden E, Fitzpatrick M, Raffaele Mendez L, Berkman K. “Different but connected”: Participatory action research using Photovoice to explore well-being in autistic young adults. Autism: The International Journal of Research & Practice. 2020;24(5):1246–59.  5. Dee-Price BJM, Hallahan L, Nelson Bryen D, Watson JM. Every voice counts: exploring communication accessible research methods. Disability & Society. 2021;36(2):240–64.  6. Heerings M, van de Bovenkamp H, Cardol M, Bal R. Burden of support: a counter narrative of service users’ experiences with community housing services. Disability & Society. 2022;1–24.  7. Cluley V. Using photovoice to include people with profound and multiple learning disabilities in inclusive research. British Journal of Learning Disabilities. 2017;45(1):39–46.  8. Watchman K, Mattheys K, Doyle A, Boustead L, Rincones O. Revisiting Photovoice: Perceptions of Dementia Among Researchers With Intellectual Disability. Qualitative Health Research. 2020;30(7):1019–32.  9. Wass S, Safari MC. Photovoice—Towards Engaging and Empowering People with Intellectual Disabilities in Innovation. Life (2075-1729). 2020;10(11):272.  10. Tajuria G, Read S, Priest HM. Using Photovoice as a method to engage bereaved adults with intellectual disabilities in research: listening, learning and developing good practice principles. Advances in Mental Health & Intellectual Disabilities. 2017;11(5/6):196–206.  11. Carawan LW, Nalavany B. Using photography and art in concept mapping research with adults with dyslexia. Disability & Society. 2010;25(3):317–29.  12. Woolrych R. Empowering images: Using PhotoVoice with tenants with special needs. Housing, Care & Support. 2004;7(1):31–5.  13. Walton G, Schleien SJ, Brake LR, Trovato C “Cat”, Oakes T. Photo voice: A Collaborative Methodology Giving Voice to Underserved Populations Seeking Community Inclusion. Therapeutic Recreation Journal. 46(3):168–78.  14. Wilson C, Kim ES. Qualitative data collection: considerations for people with Aphasia. Aphasiology. 2021;35(3):314–33.  15. Davies J, Wilson A. “What’s happening?” Examining the mental health needs of young people with learning disabilities. Learning Disability Practice. 2006;9(5):36–7.  16. Arnold SRC, Desai A, McVilly K, Hind T, Mason B, Walsh J, et al. Towards a contribution paradigm: photovoice uncovering the contribution of people with intellectual disability. Disability & Society. 2024;1–25.  17. Smith Hill RB, Shah P, Plotner AJ, Castle M, Stinnett CV. Inclusive postsecondary education and self-determination: using photovoice to centre student voices in the United States of America. Disability & Society. 2024;1–28. |
| **No information about disability type** (n=13)   - Unclear if disability is a NDD (n=7) | 1. Aamlid C, Brownfield K. We Are Not Different; We Just Sit: A Case Study of the Lived Experiences of Five College Students in Wheelchairs. Journal of Ethnographic & Qualitative Research. 2019;13(3):155–68.  2. Noyek S, Davies C, Champagne M, Batorowicz B, Fayed N. Emotional Well-Being of Children and Youth with Severe Motor and Communication Impairment: A Conceptual Understanding. Developmental Neurorehabilitation. 2022;25(8):554–75.  3. Wilbur J, Kayastha S, Mahon T, Torondel B, Hameed S, Sigdel A, et al. Qualitative study exploring the barriers to menstrual hygiene management faced by adolescents and young people with a disability, and their carers in the Kavrepalanchok district, Nepal. BMC Public Health. 2021;21(1):1–15.  4. Gibson BE, King G, Teachman G, Mistry B, Hamdani Y. Assembling activity/setting participation with disabled young people. Sociology of Health & Illness. 2017;39(4):497–512.  5. Hajjar DJ, McCarthy JW, Benigno JP, Montgomery J, Chabot J, Boster J. Weaving participation, interaction, and technology across recreational experiences: perspectives from volunteers, caregivers, and people with complex communication needs. AAC: Augmentative & Alternative Communication. 2019;35(3):217–28.  6. Wilbur J., Poilapa R., Morrison C. Menstrual Health Experiences of People with Intellectual Disabilities and Their Caregivers during Vanuatu’s Humanitarian Responses: A Qualitative Study. International Journal of Environmental Research and Public Health. 2022;19(21):14540.  7. Wilbur J, Crow CL, Poilapa R, Morrison C. Feasibility study of a menstrual health behaviour change intervention for women and girls with intellectual disabilities and their caregivers for Vanuatu’s humanitarian responses. PLOS global public health. 2024;4(1):e0002244. |
| - Not enough information about disability type (n=6) | 1. Moriña A, López-Gavira R, Molina VM. What if we could Imagine an Ideal University? Narratives by Students with Disabilities. International Journal of Disability, Development & Education. 2017;64(4):353–67.  2. Booth T, Booth W. In the Frame: photovoice and mothers with learning difficulties. Disability & Society. 2003;18(4):431–42.  3. Rabaey P, Hepperlen R, Manley H, Ament-Lemke A. Empowering Caregivers of Children With Disabilities in Zambia: A Photovoice Study. American Journal of Occupational Therapy. 2021;75(4):1–9.  4. Masquillier C, De Bruyn S, Musoke D. The role of the household in the social inclusion of children with special needs in Uganda - a photovoice study. BMC Pediatrics. 2021;21(1):1–13.  5. Kiling I, Due C, Li D, Turnbull D. Perceptions of disability, environmental risk factors and available services among local leaders and parents of young children with disabilities in West Timor, Indonesia. Disability & Rehabilitation. 2019;41(20):2421–32.  6. Burke J. ‘Some kids climb up; some kids climb down’: culturally constructed play-worlds of children with impairments. Disability & Society. 2012;27(7):965–81. |
| **Not a NDD (n=7)** | 1. Bislick L, Dietz A, Susan Duncan E, Garza P, Gleason R, Greg Kersey DH 6, et al. Finding “Zen” in Aphasia: The Benefits of Yoga as Described by Key Stakeholders. American Journal of Speech-Language Pathology. 2022;31(1):133–47.  2. Brown K, Worrall L, Davidson B, Howe T. Reflection on the benefits and limitations of participant-generated photography as an adjunct to qualitative interviews with participants with aphasia. Aphasiology. 2013;27(10):1214–31.  3. Brown K, Worrall L, Davidson B, Howe T. Snapshots of success: an insider perspective on living successfully with aphasia. Aphasiology. 2010;24(10):1267–95.  4. Pereira V.R., Coimbra V.C.C., Cardoso C.S., Oliveira N.A., Vieira A.C.G., Nobre M.O., et al. Participatory methodologies in research with children: creative and innovative approaches. Revista gaucha de enfermagem. 2017;37(spe):e67908.  5. Riga D, Bianchi F. Assessing children’s affective perception of school squares in Lombardy, Italy. Journal of Urban Design. 2024;29(6):669–89.  6. Kovačič T, Forkan C. “They Give Us That Equal Kind of Level Playing Field to Do Whatever Someone in a Regular School Does.” An Exploration of Second-level Students’ Experiences with an Alternative Education Programme in Supporting Their Education and Well-being in a DEIS Scho. Child Care in Practice. 2024;1–18.  7. Li S, Gulley J, Booty M, Firchow B, McGladrey ML. Using Photovoice to Improve Engagement in Community Health Assessments Addressing Behavioral Health. Journal of Behavioral Health Services & Research. 2024;1–18. |
| **Not about child or caregiver** (n=3) |  |
| - Population is other than child/caregiver (n=2) | 1. Miller AL, Kurth JA. Photovoice research with disabled girls of color: exposing how schools (re)produce inequities through school geographies and learning tools. Disability & Society. 2022;37(8):1362–90.  2. Clement MA, Lee K, Park M, Sinn A, Miyake N. The Need for Sensory-Friendly “Zones”: Learning From Youth on the Autism Spectrum, Their Families, and Autistic Mentors Using a Participatory Approach. Frontiers in psychology. 2022;13(101550902):883331. |
| - Not clear if findings are based on parent/family or clinician data (n=1) | 1. Feldner HA, Logan SW, Galloway JC. Mobility in pictures: a participatory photovoice narrative study exploring powered mobility provision for children and families. Disability & Rehabilitation: Assistive Technology. 2019;14(3):301–11. |
| **Engaged participants in therapy, not research** (n=1) | 1. Lo JWK, Ma JLC. Generating mutual support in multifamily therapy to promote father involvement and family communication quality of Chinese families of adolescents with Attention Deficit Hyperactivity Disorder: A qualitative study. Family Process. 2024;63(2):967–82. |
