# Supplementary material for: Using photovoice to engage underserved children with neurodevelopmental disorders and their caregivers in health research: a mixed methods systematic review
Source: Front Rehabil Sci. 2025 Aug 15;6:1638513. doi: 10.3389/fresc.2025.1638513 (PMC12394231; doi:10.3389/fresc.2025.1638513)
Supplement: Supplementary file 3 [file Table3.docx]

Supplementary Material Table 3. Quality Assessment of Selected Studies.

**Table No. MMAT Quality Assessment**

|  | Screening Questions | |  | Qualitative | | | | |  | Quantitative Descriptive | | | | |  | Mixed Methods | | | | |
| --- | --- | --- | --- | --- | --- | --- | --- | --- | --- | --- | --- | --- | --- | --- | --- | --- | --- | --- | --- | --- |
| Authors(s) | S.1 | S.2 |  | 1.1 | 1.2 | 1.3 | 1.4 | 1.5 |  | 4.1 | 4.2 | 4.3 | 4.4 | 4.5 |  | 5.1 | 5.2 | 5.3 | 5.4 | 5.5 |
| Borisov & Reid (2010) | Y | Y |  | Y | Y | Y | Y | Y |  | - | - | - | - | - |  | - | - | - | - | - |
| Cheak-Zamora et al. (2016) | Y | Y |  | Y | Y | Y | Y | Y |  | - | - | - | - | - |  | - | - | - | - | - |
| Cheak-Zamora et al. (2018) | Y | Y |  | Y | Y | Y | Y | Y |  | - | - | - | - | - |  | - | - | - | - | - |
| Danker et al. (2019) | Y | Y |  | Y | Y | Y | Y | Y |  | - | - | - | - | - |  | - | - | - | - | - |
| Do et al. (2024) | Y | Y |  | Y | Y | C | Y | Y |  | - | - | - | - | - |  | - | - | - | - | - |
| Eodanable et al. (2024) | Y | Y |  | Y | Y | Y | Y | Y |  | - | - | - | - | - |  | - | - | - | - | - |
| First et al. (2019) | Y | Y |  | Y | Y | Y | Y | Y |  | - | - | - | - | - |  | - | - | - | - | - |
| Ha & Whittaker (2016) | Y | Y |  | Y | Y | Y | Y | Y |  | - | - | - | - | - |  | - | - | - | - | - |
| Hellings et al. (2022) | Y | Y |  | Y | Y | Y | Y | Y |  | - | - | - | - | - |  | - | - | - | - | - |
| Howard et al. (2006) | Y | Y |  | Y | Y | C | Y | C |  | - | - | - | - | - |  | - | - | - | - | - |
| Mannion et al. (2024) | Y | Y |  | Y | Y | Y | Y | Y |  | - | - | - | - | - |  | - | - | - | - | - |
| Obrusnikova & Cavalier (2011) | Y | Y |  | Y | Y | Y | Y | Y |  | Y | N | Y | Y | Y |  | N | Y | Y | Y | Y |
| O’Hagan & Byrne (2023) | Y | Y |  | Y | Y | C | Y | C |  | - | - | - | - | - |  | - | - | - | - | - |
| Owen & McCann (2018) | Y | Y |  | Y | Y | Y | Y | Y |  | - | - | - | - | - |  | - | - | - | - | - |
| Scott-Barrett et al. (2023) | Y | Y |  | Y | Y | Y | Y | Y |  | - | - | - | - | - |  | - | - | - | - | - |
| Teti et al. (2016) | Y | Y |  | Y | Y | Y | Y | Y |  | - | - | - | - | - |  | - | - | - | - | - |
| Walker et al. (2020) | Y | Y |  | Y | Y | Y | Y | Y |  | Y | N | Y | Y | Y |  | N | Y | Y | Y | Y |
| Williamson et al. (2020) | Y | Y |  | Y | Y | Y | N | Y |  | - | - | - | - | - |  | - | - | - | - | - |

*Note*. MMAT = Mixed Methods Appraisal Tool. Y = Yes. N = No. C = Can’t Tell. The question numbers correspond to the questions of the MMAT checklist; the sections for quantitative randomized controlled trials and quantitative non-randomized were removed as there were no eligible studies which fell under those categories. S.1 = Are there clear research questions? S.2 = Do the collected data allow to address the research questions? 1.1 = Is the qualitative approach appropriate to answer the research question? 1.2 = Are the qualitative data collection methods adequate to address the research question? 1.3 = Are the findings adequately derived from the data? 1.4 = Is the interpretation of results sufficiently substantiated by data? 1.5 = Is there coherence between qualitative data sources, collection, analysis and interpretation? 4.1 = Is the sampling strategy relevant to address the research question? 4.2 = Is the sample representative of the target population? 4.3 = Are the measurements appropriate? 4.4 = Is the risk of nonresponse bias low? 4.5 = Is the statistical analysis appropriate to answer the research question? 5.1 = Is there an adequate rationale for using a mixed methods design to address the research question? 5.2 = Are the different components of the study effectively integrated to answer the research question? 5.3 = Are the outputs of the integration of qualitative and quantitative components adequately interpreted? 5.4 = Are divergences and inconsistencies between quantitative and qualitative results adequately addressed? 5.5 = Do the different components of the study adhere to the quality criteria of each tradition of the methods involved?
