# Supplementary material for: Using photovoice to engage underserved children with neurodevelopmental disorders and their caregivers in health research: a mixed methods systematic review
Source: Front Rehabil Sci. 2025 Aug 15;6:1638513. doi: 10.3389/fresc.2025.1638513 (PMC12394231; doi:10.3389/fresc.2025.1638513)
Supplement: Supplementary file 4 [file Table4.docx]

Supplementary Material Table 4. Sample Characteristics of the 18 Included Studies

| **Author(s) year**  **country** | **Population** | **Population subgroup** | **Sample size**  ***(n)*** | **Child age** | **Child gender** | **Child disability** | **Child functioning** | **Caregiver age** | **Caregiver gender** |
| --- | --- | --- | --- | --- | --- | --- | --- | --- | --- |
| Borisov & Reid (2010)  Canada | Youth | Not Reported | N=5 | 15 to 21years (17.2yrs) | 3 male;  2 female | ID (all) | Cognitive and language capabilities necessary to respond to questions, convey opinions, and recall past experiences. | N/A | N/A |
| Cheak-Zamora et al. (2016)  USA | Youth | Majority white  white: 9  other: 2 | N=11 | 18 to 23years (20.36yrs) | 7 male;  4 female | ASD (n=4)  Asperger’s (n=7) | Capable of engaging in group discussion (e.g., able to use some words). | N/A | N/A |
| Cheak-Zamora et al. (2018)  USA | Youth | Majority white  White: 9  other: 2 | N=11 | 18 to 23years (20.36yrs) | 7 male;  4 female | ASD (n=4)  Asperger’s (n=7) | Capable of engaging in group discussion sessions and individual interviews. | N/A | N/A |
| Danker et al. (2019)  Australia | Youth | Not Reported | N=16 | 13-17years (14.3yrs) | 15 male;  1 female | ASD (n=13)  Asperger’s (n=3)  ID (comorbid with ASD)  ADHD (comorbid with ASD) | Able to communicate verbally Able to take photos using devices  such as iPad, smartphone, digital camera. | N/A | N/A |
| Do et al. (2024)  Australia | Youth | Not Reported | N=6 | 12-16 years  (14.2yrs) | 2 male;  4 female | ASD (all) | Able to communicate verbally  Able to participate in individual interviews. | N/A | N/A |
| Eodanable et al. (2024)  UK | Youth | White (all) | N=8 | 12-19 years  (14.5yrs) | 6 male;  2 female | FASD (all)  (comorbid with ADHD (n=3), ASD (n=1), epilepsy (n=1), physical health conditions (n=3), and mental health conditions (n=1)) | Able to communicate verbally  Able to participate in group discussion sessions and individual interviews. | N/A | N/A |
| First et al. (2019)  USA | Youth | Majority white  White: 10  Not reported: 1 | N=11 | 18-23years (20yrs) | 7 male;  4 female | ASD (all) | Able to read and speak English Able to participate in group discussion sessions and individual interviews. | N/A | N/A |
| Ha & Whittaker (2016)  Vietnam | Children (n=3)  Youth (n=6) | Vietnamese (all) | N=9 | 10 to 17 years (13.2yrs) | 7 male;  2 female | ASD (all) | Verbal but verbal abilities varied (1 child could talk but generally did not have conversations with other people. 2 children could answer questions with prompts, but they preferred to talk about their own interests. 3 children could have limited conversations for a short time, and 3 other children are able to initiate and maintain conversation for a while). | N/A | N/A |
| Hellings et al. (2022)  Australia | Caregivers (mothers) only | 2 Mothers from rural area  2 Mothers from urban area | N=4  (case study) | 5 to 13 years (8yrs) | 4 male | ASD, FASD, epilepsy, speech delay (n=1)  ASD (n=2)  ASD and Ehlers Danlos (n=1) | 1 child has agoraphobia and is homeschooled 1 child is nonverbal 1 child has difficulty with motor planning No information provided for remaining child. | Not Reported | 4 female |
| Howard et al. (2006)  USA | 1 Youth  1 Mother | From rural area | N=2  (case study) | Youth: 12 years  Mother: Not reported | 1 male | Asperger’s | High functioning adolescents with Asperger syndrome with some previous social skills training (that's all that is reported). | Not Reported | 1 female |
| Mannion et al. (2024)  Ireland | Youth | Not Reported | N=13 | 12 to 18 years  (no data avail to calculate average) | Not Reported | ID (all) | Varied verbal ability (some youth struggled with verbal communication). | N/A | N/A |
| Obrusnikova & Cavalier (2011)  USA | Children (n=11)  Youth (n=3) | 12 white,  1 Asian,  1 Filipino | N=14 | 8 to 14 years (10.6yrs) | 12 male;  2 female | ASD (n=1)  Asperger’s (n=10)  PDD- not specified (n=3) | Verbal skills to communicate with the researchers and  ability to use a digital camera. | N/A | N/A |
| O’Hagan & Byrne (2023)  UK | Youth | Not Reported | N=9 | 11 to 15 years (13yrs) | 6 male;  3 female | ASD (all) | Verbal. | N/A | N/A |
| Owen & McCann (2018)  Australia | Caregivers | 1 caregiver was a single parent  6 caregivers in two-parent households | N=7 | 7 to 23 years (12.6yrs) | 6 male;  3 female | ASD (n=4)  Asperger’s (n=2)  Severe ASD and high functioning Asperger’s (n=1) | Ranged from 'mild' and 'high functioning' to 'severe' and 'profound' autism. | Not Reported | 1 male; 6 female |
| Scott-Barrett et al. (2023)  UK | Children | 3 of 12 children had English as a second language | N=12 | 7 to 11 years (no data avail to calculate average) | 9 male;  3 female | ASD (all) | All children are verbal (used spoken and written  language as their primary modes of communication at  school) | N/A | N/A |
| Teti et al. (2016)  USA | Youth | Majority white  White: 10  Asian: 1  Youth were from rural and urban areas | N=11 | 18 to 23 years (20yrs) | 7 male;  4 female | ASD (all) | Being able to read and speak English and  being capable of engaging in required study activities, as measured by young adult- and caregiver-reported  functional ability.  3 of the participants required assistance to complete the demographic survey, which may  serve as a proxy for lower functioning. | N/A | N/A |
| Walker et al. (2020)  USA | Youth (n=7) Caregivers (n=8) | From rural remote community | N=15 | 14 to 21 years (no data avail to calculate average) | 4 male;  3 female | Cerebral palsy (all) | Youth participants included a variety of functional levels  (Gross Motor Function Classification System Levels  I-IV). All children were able to take a photograph using a digital device & could communicate verbally or with an assisted device. | Not reported | 8 female |
| Williamson et al. (2020)  USA | Young adults (n=4)  Caregivers (n=4) | Indigenous (Native American) | N=8 | 19 to 24 years (no data avail to calculate average) | 4 male | Intellectual and/or Developmental Disabilities (all) | Not Reported. | 38 to 63 (no data avail to calculate average) | 1 male; 3 female |

Note. ID= Intellectual Disability. ASD = Autism Spectrum Disorder. ADHD= Attention Deficit Hyperactivity Disorder. FASD= Fetal Alcohol Spectrum Disorder. PDD= Pervasive Developmental Disorder. For funding or conflict of interest details for each study, please consult the original publications.
