# Supplementary material for: Using photovoice to engage underserved children with neurodevelopmental disorders and their caregivers in health research: a mixed methods systematic review
Source: Front Rehabil Sci. 2025 Aug 15;6:1638513. doi: 10.3389/fresc.2025.1638513 (PMC12394231; doi:10.3389/fresc.2025.1638513)
Supplement: Supplementary file 5 [file Table5.docx]

Supplementary Material Table 5. Methodological Characteristics, Funding Source, and Conflicts of Interest for the 18 Included Studies.

| **Author(s)**  **Year**  **Country** | **Recruitment setting** | **Research Design**  **& Methodological approach** | **Data collection**  **methods** | **Data analysis**  **approach** | **Funding Source** | **Conflicts of Interest** |
| --- | --- | --- | --- | --- | --- | --- |
| Borisov & Reid (2010)  Canada | Educational setting (segregated school: physical education classes; intramural sports league; swimming program) | Qualitative  Interpretive Phenomenology | Field observations, video recording, photos and semi-structured interviews | Interpretive Phenomenological Analysis | Not Reported | Not Reported |
| Cheak-Zamora et al. (2016)  USA | ASD clinic | Qualitative  Qualitative Description (no specific method) | Photovoice and semi-structured interviews | Thematic Analysis | The Richard Wallace  Faculty Incentive Grant | No Conflicts of Interest Declared |
| Cheak-Zamora et al. (2018)  USA | ASD clinic | Qualitative  Qualitative Description (no specific method) | Photovoice and semi-structured interviews | Thematic Analysis | The Richard Wallace  Faculty Incentive Grant | No Conflicts of Interest Declared |
| Danker et al. (2019)  Australia | Educational setting (high school) | Qualitative  Grounded Theory | Photovoice and semi-structured interviews | Grounded Theory | Authors state they did not receive funding for the research | Not Reported |
| Do et al. (2024)  Australia | Community (community orgs’ websites, social media platforms, newsletters) | Qualitative  Qualitative Description (no specific method) | Author reflection  Participant feedback | Not Reported | Deakin  University | No Conflicts of Interest Declared |
| Eodanable et al. (2024)  UK | Community, website, social media | Qualitative  Interpretive  Phenomenology | Individual interviews  Group meetings | Interpretive Phenological Analysis | No external funding was received | Not Reported |
| First et al. (2019)  USA | Community (ASD centre) | Qualitative  Phenomenology | Photovoice and individual interview after public exhibit | Phenomenological analysis approach | The Richard Wallace  Faculty Incentive Grant Program, University of Missouri | No Conflicts of Interest Declared |
| Ha & Whittaker (2016)  Vietnam  Australia | Community organization serving children with ASD and a parent-run school for children with ASD | Qualitative  Qualitative Description (no specific method) - Authors note this study was part of a larger ethnographic study | Photovoice (PV was used as part of a broader ethnographic study which included participant observation, in-depth interviews with parents, caregivers, and health professionals, an online survey, and a public exhibit. For this paper, only PV was used) | Content analysis | 1.International Postgraduate Research Scholarships;  2.The University of Queensland;  3.The Organization for Autism Research | No Conflicts of Interest Declared |
| Hellings et al. (2022)  Australia | Community organization (where dog was obtained) | Qualitative (multiple descriptive case studies)  Interpretive Phenomenology | Photovoice and semi-structured interviews conducted pre and post dog placement | Interpretive Phenomenological Analysis | No financial support was received | No Conflicts of Interest Declared |
| Howard et al. (2006)  USA | Not Reported | Qualitative (case study)  Grounded Theory | Photographs, semi-structured interviews, standardized scales | Grounded Theory | Not Reported | Not Reported |
| Mannion et al. (2024)  Republic of Ireland | Education  (mainstream post primary schools) | Qualitative  Qualitative Description (using Transformative paradigm) | Meetings | Reflective Thematic Analysis (informed by initial analysis conducted by participants and researchers) | Mary Immaculate College, Research and Graduate School | No Conflicts of Interest Declared |
| Obrusnikova & Cavalier (2011)  USA | Community organization serving children with ASD | Mixed Methods  Qualitative Description (no specific method) | Photovoice, semi-structured interviews, standardized scales, accelerometry data, activity logs | Univariate Descriptive Analysis  Qualitative analysis followed a 3 stage process (open coding, assessing intercoder reliability, organizing the codes into six levels of the socioecological model) | This work was partially supported by grant funding from IM Able Foundation | Not Reported |
| O’Hagan & Byrne (2023)  UK | Autistic Networks, Special Education Networks, Main stream and special education primary schools, Online platforms (Facebook, Twitter | Qualitative  Qualitative Description (no specific method) | Photovoice and Zoom interviews | Thematic Analysis | Queen’s University Belfast | No Conflicts of Interest Declared |
| Owen & McCann (2018)  Australia | Community (autism support organization)  Autism advisors, local radio | Qualitative  Narrative Research | Photovoice and in-depth interviews | Framework Analysis | University of Tasmania Research Enhancement Grant Scheme | No Conflicts of Interest Declared |
| Scott-Barrett et al. (2023)  UK | Educational setting (mainstream primary school) | Qualitative  Qualitative Description (no specific method) | Photovoice, semi-structured interviews, walking tours, LEGO model building | Thematic Analysis | University of Edinburgh Principal’s Career Development  Scholarship | No Conflicts of Interest Declared |
| Teti et al. (2016)  USA | Community -ASD Research Centre | Qualitative  Qualitative Description (no specific method) | Photovoice, individual reflection interview | Thematic Analysis | The Richard Wallace  Faculty Incentive Grant Program, University of Missouri | Not Reported |
| Walker et al. (2020)  USA | Not Reported | Mixed Methods  Qualitative Description (no specific method | Photovoice, in-depth interview, parent focus group, quantitative measures | Univariate Descriptive Analysis  Content Analysis | Not Reported | No Conflicts of Interest Declared |
| Williamson et al. (2020)  USA | Community organizations serving Native American Individuals with Intellectual and/or Developmental Disabilities (IDD) | Qualitative  Qualitative Description (no specific method) | Photovoice, semi-structured interviews | Used socio-ecological model to frame the results (used model levels as categories) | 1.American Occupational Therapy Foundation  2.Northern Arizona University Faculty Grant Program | No Conflicts of Interest Declared |

*Note:* ASD = Autism Spectrum Disorder. IDD = Intellectual and/or Developmental Disabilities.
