# Supplementary material for: Using photovoice to engage underserved children with neurodevelopmental disorders and their caregivers in health research: a mixed methods systematic review
Source: Front Rehabil Sci. 2025 Aug 15;6:1638513. doi: 10.3389/fresc.2025.1638513 (PMC12394231; doi:10.3389/fresc.2025.1638513)
Supplement: Supplementary file 6 [file Table6.docx]

Supplementary Material Table 6. Health Research Areas that have Used Photovoice When Population Under Study are Children with NDDs and their Caregivers.

| Area of health research | Study focus | Study authors |
| --- | --- | --- |
| Education Settings | Experience of being a tutor or teacher assistant in physical education (students with intellectual disabilities) | (65) |
|  | The experience of autistic young people in secondary education settings during the first COVID-19 lockdown | (80) |
| Physical Activity Participation | Perceptions of barriers and facilitators to after-school participation in physical activity (children with ASD) | (81) |
|  | Barriers and facilitators to physical activity participation (youth with CP from rural areas) | (82) |
| Transitioning into adulthood | Experience of transitioning into adulthood (youth with ASD) | (66) |
|  | Experience of transitioning into adulthood (youth with ASD) | (67) |
|  | Perceptions of stress/coping with transitioning into adulthood (young adults with ASD) | (70) |
|  | Experience of growing up and transitioning into adulthood (young adults with ASD) | (76) |
| Health and Wellness | The experience of health and wellness (Native American adults with IDD and their caregivers) *young adults | (77) |
|  | Perspectives of wellbeing (conceptualization, barriers, ways to enhance) (students with ASD) | (68) |
|  | The experience, health needs, and well-being of children with ASD | (71) |
| Area of health research | **Study focus** | **Study authors** |
| Life Experience (friendship, assistance dog ownership, housing adaptations) | Lived experience of adolescents with FASD | (69) |
|  | The experience of friendship (adolescent with Asperger syndrome). | (79) |
|  | The life, experience, and health needs of young people with ASD | (71) |
|  | Experience of assistance dog ownership on families of children living with autism | (72) |
|  | Impact of housing adaptations for families living w a child on the autism spectrum | (74) |
| Approaches to Meaningfully Engage Population of Interest | Approaches that can be used to meaningfully engage autistic children in research | (75) |
|  | To explore the process of using online photovoice (benefits & challenges) to facilitate the participation of autistic adolescents in a research study | (78) |
|  | To detail how photovoice (in-person) was used to support students with intellectual disabilities to voice their experiences & perspectives in mainstream post-primary schools | (73) |

*Note:* NDD= Neurodevelopmental Disorder. ASD = Autism Spectrum Disorder; CP = Cerebral Palsy; IDD = Intellectual

or Developmental Disability; FASD=Fetal Alcohol Spectrum Disorder.
