# Supplementary material for: Using photovoice to engage underserved children with neurodevelopmental disorders and their caregivers in health research: a mixed methods systematic review
Source: Front Rehabil Sci. 2025 Aug 15;6:1638513. doi: 10.3389/fresc.2025.1638513 (PMC12394231; doi:10.3389/fresc.2025.1638513)
Supplement: Supplementary file 7 [file Table7.docx]

Supplementary Material Table 7. Contextual Considerations When Using Photovoice with Children with NDDs and their Caregivers

| **Category** | **Representative Quotation** | **Study Authors** |
| --- | --- | --- |
| Using Photovoice Required Flexibility  (16instances) | *“Flexibility was required to address disruptions caused by home environments, such as external distractions and the presence of parents during sessions, while ensuring the autonomy of participants in the image creation and discussion process.” (78)* | (65, 68, 69, 71, 77, 78, 81) |
| Using Photovoice Required More of Everything  (e.g., support, time, patience)  (15instances) | *“Some participants [young adults with ID] required more support to successfully use their cameras and others required regular visits to the family home to maintain communication about the project.”* *(77)* | (68, 69, 71, 73, 77, 78, 82) |
| Unique Engagement and Analysis Challenges  (11instances) | *“In participatory action research in general and photovoice, analysis is a process of engagement between researchers and research participants…the communication challenges related to ASD often made such analysis challenging.” (71)* | (68, 78, 71, 73) |
| The Functioning Level of NDD Children and Youth Influenced Level of Contribution  (8instances) | *“The language skills of students were an inhibiting factor in describing photographs or the meaning behind them.” (73)* | (65, 66, 71, 73) |
| Asking Consent to Take Photos of Others and Complying with Photo Instructions was Challenging for NDD Children/Youth  (6instances) | *“Our initial instruction for children to photograph particular topics was not workable and instead children freely photographed whatever they wished.”*  *(71)* | (71, 73, 81) |

| **Category** | **Representative Quotation** | **Study Authors** |
| --- | --- | --- |
| NDD Children and Youth Feared Participating in Groups and Breaking the Rules of Photo-Taking in School  (4instances) | *“Youth [ASD] described apprehension about talking in a group-setting and worried their pictures would be criticized.” (66)* | (66, 68, 71) |

Note. NDD = Neurodevelopmental disorder; ASD = Autism Spectrum Disorder
