# Supplementary material for: Using photovoice to engage underserved children with neurodevelopmental disorders and their caregivers in health research: a mixed methods systematic review
Source: Front Rehabil Sci. 2025 Aug 15;6:1638513. doi: 10.3389/fresc.2025.1638513 (PMC12394231; doi:10.3389/fresc.2025.1638513)
Supplement: Supplementary file 8 [file Table8.docx]

Supplementary Material Table 8. Adaptations Made When Using Photovoice with Children with NDDs and their Caregivers.

| **Nine Recommended Photovoice Steps**  **(Wang & Burris, 1997)** | | **Adaptation** | **Study authors** |
| --- | --- | --- | --- |
| 1. Group Size for  Photovoice  (7 to 10 people is an ideal group size) | | - Conducted Individually | (65, 68, 69, 72, 74,78, 79, 80) |
|  |  | - Conducted in small groups and/or individually | (66, 67, 69, 71, 75, 76, 81) |
|  |  | - Conducted in groups of 7+ | (69, 70, 77, 82) |
|  |  | - No information provided re: group size | (73) |
|  | |  |  |
| 2. Select a Target  Audience of Decision-Makers (Participants select this group) | | - Researchers selected stakeholders | (68, 69, 80) |
|  |  | - No information provided on target audience | (65, 66, 67, 71, 72, 74, 75, 76, 77, 78, 79, 81) |
|  |  | - No adaptations reported   (participants selected target audience) | (70, 73, 82) |
|  |  |  |  |
| 3. Obtaining Informed Consent  (Researcher must obtain consent from each participant) | | - Adapted information sheet & consent form - Adaptations of consent forms included accessible (simplified, easy to read, child-friendly) language and use of visuals. - To ensure participants’ understanding of consent and/or assent, adaptations included discussions between parents and children, asking participants for feedback as the research steps were explained, and using teach-back questions to check understanding. - A short video illustrated the project purpose, participant roles, & right to withdraw. - Emotional wellbeing check-ins were provided throughout the research. Standard procedure for addressing potential emotional upset during sessions was developed & information on local support organizations was shared with individuals and parents. - A break card was provided to indicate needing time or space during the research process. | (66, 68, 69, 71, 73, 75, 77, 78, 80) |
|  |  | - Ongoing assent sought at each project phase. - Non-verbal & visible ways for children to indicate ongoing assent was provided, including traffic light cards (green: continue, orange: pause, red: end the research encounter) and smiley faces (smiley face = want to participate; sad face = do not want to participate). | (69, 73, 75) |
|  |  | - No adaptations reported - No information provided on obtaining informed consent. | (65, 72, 81, 82)  (67, 70, 74, 76, 79) |
|  | |  |  |
| 4. Introduce Photovoice to Participants  (Researcher introduces methodology & facilitates discussion about camera and ethics) | | - In a group setting and/or individually with adaptations - The facilitator explained the purpose of the project, discussed the ethics of picture-taking (i.e., inappropriate pictures; obtaining consent to take a person's picture), and taught participants how to use their cameras. Participant also had the opportunity to get to know each other. - Parents and children attended separate group training sessions to ensure each participated independently and each perspective was documented. - A simple introduction about the cameras was given. - Children took photos by themselves for about 20 minutes. A handout with simple language and pictures was also given to children. - Depending on the child’s level of attention, the sessions were conducted individually or in a small group. - An illustrated study information booklet was used to explain the research process. - PowerPoint slides were used to explain the study’s purpose, what participation entailed, how to take photographs and transfer them to the computer, and rules of taking photographs in school. - Samples of Photovoice project were shown, printed, & given to participants. | (67, 68, 71, 75, 76, 81, 82) |
|  | | - Online and via PowerPoint presentations emailed to participants - Presentation slides were used as visual aids to communicate the study’s purpose and explain the research process. - Regular communication and continuity was maintained through ongoing correspondence with participants via emails and texts (re: address questions, summarize previous discussions). - Parents provided technological support, reminders for sessions, and accompanied the young people in initial meetings. Parents were welcome to attend sessions in line with their child’s wishes. - School staff were asked not to influence students’ choice of photographs but could assist, if necessary, with holding the iPad and with recording on the prompt sheet when photos were taken. Staff provided verbal reminders to participants to take photos each day. - To establish rapport & relationship with participants, a short semi-structured interview was used to gather details about likes, dislikes, subjects, and challenges students encounter in school. The wording of questions was adapted to support students. A booklet was created where questions were presented visually using emojis. Students had the opportunity to draw, write or say, their answers. - Participants were given a checklist of prompts for suitable photographs they may wish to take. Prompts and guidance on taking photos were accessible and child-friendly. - Adaptations were made to the process throughout the various stages to enable student engagement and included: extra visits to work with   students, incorporating additional supports to aid communication (use of written, digital, visual and verbal methods), reducing the number and type of photos students could take.   - Efforts to involve students in developing the research questions were incorporated throughout (e.g., asking students if there were aspects of their school experience they’d like to explore). - Three PowerPoint presentations were emailed to participants to guide them through the project [conducted during COVID-19]: (1) To introduce the research team, explain the purpose of the study, and introduce the concept of Photovoice, (2) to provide a short accessible guide to documentary photography, and (3) to explain the research questions and detailed instructions of the Photovoice task. | (73, 78, 69, 80) |
|  | | - No adaptations reported | (66, 70, 77) |
|  | | - No information provided on how participants received information (re: photovoice methodology, the camera, and ethics). | (65, 72, 74, 79) |
| 5. Brainstorm with Participants | | - Researcher Chose the Topic (gave participants photo topic) | (65, 67, 68, 69, 71, 73, 74, 78, 79, 80) |
| (participants brainstorm together about photo topics/themes. Participants may discuss ideas of what photos to take) | | - Various ways of identifying photo topics - Researchers chose the topic and participants brainstormed photo ideas as a group or individually. - Youth and their parents attended separate brainstorming sessions to brainstorm topics for taking photos. - Participants took photos of anything they wanted. - Using a game called Talk-n-Toss, participants identified the focus of their photos in relation to predetermined topics. | (66, 70, 75, 77, 81, 82) |
|  | | - No adaptations reported | (76) |
|  | |  |  |
| 6. Distribute Cameras | | - Used disposable or digital camera | (65, 66, 67, 70, 71, 76, 77, 81) |
| (Have used: autofocus cameras, disposable camera, medium focus Holga cameras, digital cameras) | | - Used own devices, tablet, or other creative possibilities - Participants used their devices (i.e., digital cameras, smartphones and/or tablets) to take photos. - Participants were provided with tablets to take photos for ease of use. - Participants used drawings, personal photos or images found online or in magazines. | (68, 78, 73, 75, 80, 82) |
| 7. Provide Time to | | - Given ten days, two or three weeks, a month, or took photos on the spot | (69, 72, 75, 77, 78, 81, 82) |
| Take Photos (given 7 | | - No adaptations reported (had 1 week to take photos) | (66, 67, 68, 73, 76) |
| days to take photos) | | - No information provided about time given to take photos | (65, 70, 71, 74, 79, 80) |
| 8. Meet to Discuss the Photos and Identify Themes | | **Selecting and Contextualizing Photos**   - Via adapted interviews - Individual semi-structured interviews were conducted with modifications based on participants’ vocabulary and language comprehension. - The interview schedule had more complex questions at the end of each section and included open-ended questions as they allowed the same questions to be asked using different formats. - Online interviews were conducted, using PowerPoint to display photos and using a modified version of the SHOWed method to ask questions (What is the story behind this photo? What does this photo show about your life as a person with FASD?). - Pictures from the iPad were used as visual prompts for the interview. Students were asked to narrate the contents of the photos and were supported in this process by the PI asking five questions developed by the researchers (e.g., Describe your picture, What is happening in our picture? Why did you take a picture of this? ) - Participants brought their favourite photos to share and discuss in an interview; the photographs acted as prompts for the discussion. - Tablets were used to display photos as visual prompts to answer pre-determined questions; A Placement Graphic Organiser (Bennett & Rolheiser, 2001) and sentence starters were used to support the narrative process. - An online questionnaire was used in addition to individual interviews. Researchers supported participants in the completion of the questionnaire (e.g., rephrasing words the participants did not understand, helping participants stay on task via reminders and prompts, reminding participants to respond based on their feelings/thoughts). Modifications were used for those who needed them (e.g., completing the questionnaire during one or various administrations, using a two-point scale rather than a four-point scale to answer questions). - Online interviews were conducted on Zoom. Participants emailed their photos with short descriptions of their meaning prior to the interview. - Participants chose which photographs to discuss. Follow-up questions were asked to provide clarification or probe for deeper insights. - Pictures were projected onto a screen; Parent and child participants were interviewed separately. The SHOWeD method was used to help participants contextualize the meaning of their photos. Parents also attended a focus group to review and further contextualize the chosen photographs. The SHOWeD method was used again. | (65, 69, 72, 73, 74, 77, 79, 80, 81, 82) |
|  | | - Via adapted meetings (individual or group) - Each participant presented approximately 3 to 13 photos to the group and discussed what each photo meant; Facilitators used a semi-structured guide to encourage youth’s stories. - During an individual meeting, photographs were viewed using the interviewer’s tablet. Questions developed by the researcher were used to facilitate discussion (e.g., ADD). - During online individual sessions with researcher, images selected by the participants were discussed. Participants used the share-screen feature to present and discuss photos. The researcher used a conversational approach using verbal prompts instead of structured questions (e.g., Can you talk a bit more about this image? Can you say why you drew this picture?) - During group meetings, a laptop was used to download digital photos and display them via a projector. The facilitator provided prompt***s*** for discussion (e.g., What does this photo capture about your life? What does the picture mean to you?) - During group or individual meetings, participants were asked to choose photos that they wanted to print. Participants made albums using the photos and craft materials and were asked about the photos. Participants described whatever they wanted about their photos. Simple prompt questions were used (e.g., Tell me about your photos? Who was in the photo?) - Information obtained through interviews with the children/youth, observations, and parents’ interviews were used to help contextualise photographs. - Discussion with the researcher about the photos took place in pairs or individually (based on class timetabling and teachers’ advice); The photographs were made into a PowerPoint presentation. Children could type captions if they wished and could control speed at which they looked through the photos. | (66, 67, 68, 70, 71, 75, 76, 78) |
|  | | **Identifying Themes**   - Researchers identified themes | (66, 70, 72, 74, 76, 77, 79) |
|  | | - Researchers identified themes with member checking or project advisory identified themes - During an interview with each participant, the researcher shared a tentative summary of the findings and asked participants if the themes made sense. - Researchers created a codebook describing the most salient themes. The photo exhibit served as a form of “member checking” where participants checked on data interpretation, reviewed and discussed major analysis findings for dissemination, and assured that these findings represented their experiences. - Transcripts were shared with participants for member-checking to verify the accuracy of the interpretation of their discussions. - A feedback session was conducted five months later to seek the participants’ feedback on the analysis of the data. Sessions took place during class time and children were consulted in pairs. Researchers made the session as accessible as possible by using minimal text or writing. PowerPoint presentations with photos taken by each pair of children were used to prompt children’s recollection of participating. - Participants (parents & adolescents) identified common themes in the photos. - Young advisors led a discussion on how each photograph could be thematically categorised. | (65, 67, 75, 78, 81, 80, 82) |
|  | | - Participants not involved in identifying themes | (68, 71) |
|  | | - Both researchers and participants were involved with adaptations - In an online group meeting, participants were guided by the researcher in analysis of their selected photos. First, the group identified labels for each photo and then codes (what, who, where). Then they were supported to develop categories. The researcher used Google Jamboard to visually represent labels and descriptive categories during the discussion. - Participants were given a copy of their photographs and were asked to select which pictures best told their story; With the support of the researcher, participants grouped printed photographs into two categories. - The analysis undertaken with participants informed the analysis conducted by researchers - A focus group interview with Special Education Teachers and Special Educational Needs Co-ordinators from participating schools provided a nuanced understanding of developing themes from the data collection process (provided contextual background to students’ lived experiences). | (69, 73) |
| 9. Plan a Format to | | - Not specified who chose medium | (66, 67, 68, 70, 71, 73, 76, 77, 80, 82) |
| Share Photos  (facilitator and | | - No adaptations reported (participants & researchers chose medium to share photos together) | (69) |
| participants choose the best medium to present photos) | | - No information provided on choosing medium to share photos | (65, 72, 74, 75, 78, 79, 81) |
| An Extra Step: Seeking Participant Feedback on Research Activities | | - Various ways of seeking feedback - Participant experience in the photovoice project was sought through: individual interviews, reflection questions (e.g., How did you experience participating in this study?), a feedback form. - Feedback questions were developed using simple language. Children could also respond using emojis. | (66, 67, 78, 69, 70, 73, 75, 76) |
|  | | - Feedback not sought | (65, 68, 71, 72, 74, 77, 79, 81, 80, 82) |

Note. NDD = Neurodevelopmental disorder.
