# Supplementary material for: Using photovoice to engage underserved children with neurodevelopmental disorders and their caregivers in health research: a mixed methods systematic review
Source: Front Rehabil Sci. 2025 Aug 15;6:1638513. doi: 10.3389/fresc.2025.1638513 (PMC12394231; doi:10.3389/fresc.2025.1638513)
Supplement: Supplementary file 9 [file Table9.docx]

Supplementary Material Table 9. Practicality as Reported by Authors- Photovoice as a Useful and Valuable Methodology.

| **Category** | **Sub-Category** | **Representative Quotation** | **Study Authors** |
| --- | --- | --- | --- |
| Reported  Photovoice  as Useful  and Valuable | To Help NDD  Children/Youth  and their Caregivers  Express their  Experiences  (23 instances) | *“By choosing Photovoice methodology, this study has enabled the young autistic experts to convey their experiences and concerns and has produced useful, authentic knowledge and understanding.” (80)*  *“Photovoice as a data collection tool can successfully be utilised to provide the*  *space, voice, audience and influence identified in Lundy’s Framework of*  *Participation (2007). In this sense it is ideal for use in schools for seeking the perspectives of children to facilitate change.” (73)* | (65, 67, 68, 69, 70, 71, 72, 73, 75, 76, 77, 78, 80, 81, 82) |
|  | To Empower NDD  Youth and their  Parents  (10 instances) | *“Photovoice is a suitable method for not only eliciting the perspectives of youth*  *with ASD on varying topics but also as a tool to support and empower*  *youth.” (67)*  *“Photovoice empowered youth to document their experiences through photos.”*  *(66)* | (66, 67, 71, 73, 76, 78) |
|  | To Meaningfully  Engage  Participants  Including  Underrepresented  Groups  (9 instances) | *“Photovoice proved a useful means of allowing children with ASD and their*  *parents an opportunity to describe their lived experience and engage*  *meaningfully with research.” (71)*  *“Online photovoice in this study proved to be highly feasible, as reflected in the consistent participation of young people in all phases… Therefore, photovoice’s potential to offer a more inclusive and authentic engagement with young people with FASD could be further realised.” (69)* | (66, 69, 71, 73, 78, 80) |

Note. NDD = Neurodevelopmental disorder; ASD = Autism Spectrum Disorder, FASD = Fetal Alcohol Spectrum Disorder
