# Supplementary material for: Using photovoice to engage underserved children with neurodevelopmental disorders and their caregivers in health research: a mixed methods systematic review
Source: Front Rehabil Sci. 2025 Aug 15;6:1638513. doi: 10.3389/fresc.2025.1638513 (PMC12394231; doi:10.3389/fresc.2025.1638513)
Supplement: Supplementary file 10 [file Table10.docx]

Supplementary Material Table 10. Recommendations Provided by Study Authors to Advance the Use of Photovoice with Children with NDDs and their Caregivers.

| **Category** | **Recommendation** | **Study Authors** |
| --- | --- | --- |
| Implementing the Photovoice Methodology in Research | **Strategies for Implementing Photovoice**   - Have an individual introduction session and interview prior to having photovoice group sessions. - Use point-and-shoot cameras with automatic flash to make it easier for participants to take photos. - Allow participants to keep the camera. - Have designated times to meet and share photos. This may help participants share photos in a timely manner. - Work one-on-one with participants with ASD as they may find groupwork (e.g., to present/discuss photos, to identify themes) stressful due to their communication issues. - Provide more examples of acceptable vs unacceptable photos to allay fears of unintentionally breaking the rules by taking inappropriate photographs in school. - Identify advocates within the organization/group you are working with who are likely to be invested and have time to contribute to the research (identify those who have time, commitment, & interest in the study) - Collaborate more closely with advocates (e.g., teachers, parents) and get their support in reminding students to take photographs - Be adaptable and resourceful when conducting online photovoice studies, particularly in contexts with limited technological resources and unstable internet connections - Anticipate and address challenges proactively to enhance the overall quality and effectiveness of the research process - Ensure the comfort of autistic adolescents throughout their research participation. Create a comfortable and relaxed environment for the productive and ethical conduct of research with autistic young people. - Parental support should be explicitly addressed at the outset of research - Parental support was invaluable due to the nature of young people’s executive functioning, as parents organised and aided attendance, and ensured IT access to the online meetings - Researchers need to consider what happens if students are absent on the day of   arranged meetings. In this study, a way to circumvent this involved the school  contacting the PI, if a child was absent. | (66, 68, 69, 73, 78) |
|  | **Using Interviews with Photovoice**   - Semi-structured interviews allow the researcher to establish rapport, provide   Opportunities to ask questions, use probes when necessary, and follow interests and  concerns originating from the participant. (youth with ID)   - Students with an intellectual disability can be interviewed. Specific strategies included   multiple data collection procedures, rephrasing of questions, follow-up questions and  probes, using open-ended questions, obtaining teacher input, allowing time for repetition and comprehension, and careful phrasing format.   - [Participant’s name]’s showed interest in the interview process and thoughtful responses. Interviews may be a useful means to connect with adolescents with Asperger syndrome. - When using interviews and photovoice, pointing to the photos taken and asking   questions about the photos can help interviewers’ direct students with ASD back to the  interview topic. | (65, 68, 79) |
|  | **Photovoice and Data Analysis**   - Children should be “active agents in interpretation. Analysis in Photovoice studies should take a two-step approach: analysis where children are co-creators of meaning and analysis undertaken by the researcher which is informed by the first step - Important to acknowledge the voices of the children when interpreting photographs and not to impose our own categories of interpretation, whether these are from biomedicine or assumptions about children’s abilities. - Avoid making assumptions about photographs taken by children with autism (e.g., interpreting photos as product of autistic fixations etc.). - The intention with which these photographs were taken and their aesthetic values remind us that these are artistic expressions and forms of communication, not only photographs by children with autism or products of their disabilities. - Incorporate triangulation (e.g., with observations, interviews with others other than the children) when interpreting photographs as this will provide greater understanding of how/why children take and choose photographs. - Integrating the perspectives of special education teachers and special educational needs coordinators provided contextual background to students’ lived experiences, deeper insight, and clarity - Using a phenomenological case study approach with photovoice when have a small sample size can be useful in generating rich data/insights. - Familiarisation of data is a crucial step in Photovoice studies and should coincide with data collection. Prior to meeting each participant for the next phase of the Photovoice process, interviews were listened to and transcripts, fieldnotes, and journal entries were read. This is important as it allowed the PI to familiarise herself with the data and to get to know the participants. - To reduce PI’s interpretation of responses to interview questions (e.g., assumptions), it is important to get to know participants. | (71, 72, 73) |
|  | **Online Photovoice to Expand Recruitment Reach**   - The implementation of online photovoice in this research allows the inclusion of geographically dispersed autistic adolescents who would otherwise have been unreachable - By leveraging online platforms, researchers would not be constrained by geographical limitations and could expand their recruitment reach beyond their local area, promoting larger community involvement - The use of online photovoice in this study facilitates the recruitment and engagement of participants from both urban and rural areas - The use of online photovoice allows for wider representation instead of a smaller geographical focus | (69, 78) |
| Use in Practice Settings | **Help NDD Children/Youth Express their Experiences, Perspectives, and Needs**   - Clinicians can explore the usefulness of photography and creative art forms as tools to help youth with ASD voice their challenges and solutions. - Clinicians can use photovoice in practice interventions to overcome communication issues and provide youth with a tool to voice their experiences. - Clinicians can explore ways to use images to improve adults with ASD's expressive and receptive communication skills and health literacy. - Photovoice may be used to communicate with, take care of, and provide medical care for this vulnerable population (youth with ASD). - Smartphones and photo-sharing applications can facilitate using photovoice in practice and treatment-based interventions to overcome communication issues in youth with ASD. - Photovoice can be a valuable tool to determine the acceptability and validity of interventions used for people with ASD in education, public health, and social policy. - Photovoice can be used as a tool to facilitate motivation and empowerment and help young adults with ASD express their experiences and needs in practice settings. | (66, 70, 71, 76) |
|  | **Facilitate Engagement of NDD Youth and Families in Community Activities, Program/Policy Development and Therapy**   - Photovoice may be used be as a tool to engage students in counselling and therapy. - Engaging young adults through photos could allow them, regardless of functioning level, to participate in group or community activities. - When designing ASD programs, services, and policies, photovoice can be used as a tool to help youth with ASD voice their concerns and solutions. - Incorporate more opportunities for families, children and adults with CP and other mobility impairments to develop specific programing to ensure accessibility of programs, equipment, and public spaces. | (68, 70, 76, 82) |
| Facilitating  Meaningful  Engagement in  Research | **Use Diversity of Data Collection Approaches and Adapt as Needed**   - Provide participants with multiple options for creating and selecting images for research purposes - Provide the opportunity to capture new photographs and use existing ones - Use visual methods and diverse approaches to cater to the communication preferences of participants, such as talking, drawing, and writing independently or with assistance - Offer a wider range of communication alternatives, such as written expression or pictograms, to include participants with non-verbal abilities - Expanding the range of creative possibilities beyond the traditional camera-based approaches (e.g., drawings, internet images, personal photo collections), encouraged the adolescents to exercise autonomy in selecting the issues they wished to discuss and determining how they would visually represent their experiences. - A flexible, mediated approach must be prioritised to provide opportunities to participate and adjust the processes as necessary for research to be truly inclusive. - A multimodal approach to data collection can create opportunities for different modes of expression allowing for creative communication without having to rely just on one expressive form. - A multimodal approach to data collection would ensure the participation of children of various abilities (e.g., nonverbal as well as verbal). - Develop a diversity of approaches so that autistic young people can share and explain their ideas, experiences and views in ways that suit their communicative preferences and interests. - Researchers must develop multiple meaningful ways to listen to the views of autistic children and young people as different methods may be helpful. - Use a flexible research design with multiple opportunities for self-expression through different modes (which can be adapted to suit individuals’ strengths and needs). - Adapt data collection approaches to be respectful of individuals preferred communication modes and to be responsive to the communicative cues and wishes of those involved. - Adapt research activities to ensure they are worthwhile, interesting, and valuable to participants. - Research activities and foci should be relevant and meaningful to research participants. This may result in multiple research foci as the participants direct researchers’ attention to elements that are important to them and their lives. | (73, 75, 78) |
|  | **Attend to Power Dynamics, Communicate Respect, and Recognize Expertise**   - Acknowledge power dynamics when exploring children’s self-expression and engagement with different activities: especially in terms of the way power differentials between children/adults and researcher/participant can determine who controls what is discussed or created and can highlight differences in knowledge and expertise. - Attend to power differentials in research (e.g., applying ethical reflexivity, reflecting on where attention of the researcher and the group is being directed & how it can exacerbate/ameliorate the power dynamic in research encounters) to facilitate meaningful and respectful research interactions. - All elements of research (e.g., research practices, processes) should communicate sincere respect to participants involved. - Recognize participants’ authority, expertise (lived experience) and insights on research methods and processes. A helpful way of integrating this is ensuring children are given the opportunities to co-design, adapt, comment on, take control of and/or feedback on methods. - Adopt a strength-based approach (as opposed to deficit framing) where participant strengths are recognized and valued. - Acknowledge and communicate that the participants’ contributions are valued and valuable. | (75) |
|  | **Co-create Methods and Research Foci**   - Modify and adapt online photovoice to set the stage for collaborative, empowering research practices in which online photovoice becomes a powerful tool for autistic youth to actively contribute to the discourse surrounding their lives. - Children are co-creators of meaning and analysis undertaken by the researcher - Provide opportunities to co-design, adapt, comment on, take control of and/or feedback on methods as this is an important step in finding ways for meaningful communication and interaction. - Find ways to listen to and learn about autistic school experiences, to collaboratively co-construct evidence and to include autistic children in educational research and decision-making. - Co-creation of research from the outset can facilitate greater ownership and engagement. - Create opportunities for deeper - participation in the co-creation of methods and research foci. - Open dialogue about the focus and methods of research with autistic people and their allies can help contextualize the work and foster participant learning about the research process. | (73, 75, 78) |
|  | **Provide Ownership** **of Research Processes/Materials**   - Give participants choice as to photos they would like to use and visual they would like to create. Choice and flexibility in Photovoice studies empower participants and reposition power and control throughout the research process - The more control and power handed over to the children (e.g., give control over the voice recorders, tablet, photographs to take), the more meaningful the processes and the more engaged they may be with the process. - By handing over control and power to children (e.g, giving the children control over the voice-recorders, tablet), researchers offer opportunity to engage, to creatively use the equipment, and to focus the researcher’s attention on what children perceive as important. - Support autistic children’s ownership of research materials and processes to support their engagement in the research. This handing over of power includes handing over of voice-recorders or cameras, or allowing the children to choose the topic of discussion, critique the methods, or ask questions rather than just respond to them. | (73, 75) |
|  | - Offer children choice over how they wish to express themselves, flexibility over the pace (and if possible, topic) of research interactions, and opportunities to express choice and expertise. - Give children participating in Photovoice choice as to whether they would like to work in pairs or individually. |  |

Note. NDD = Neurodevelopmental disorder; ASD = Autism Spectrum Disorder, ID = Intellectual Disability, CP = Cerebral Palsy
