# Supplementary material for: Using photovoice to engage underserved children with neurodevelopmental disorders and their caregivers in health research: a mixed methods systematic review
Source: Front Rehabil Sci. 2025 Aug 15;6:1638513. doi: 10.3389/fresc.2025.1638513 (PMC12394231; doi:10.3389/fresc.2025.1638513)
Supplement: Supplementary file 11 [file Table11.docx]

Supplementary Material Table 11. Future Research Questions Suggested by Study Authors to Advance the Use of Photovoice with Children with NDDs and their Caregivers.

| Finding | Category | Sub-category | Suggestion | Study authors |
| --- | --- | --- | --- | --- |
| Future Research Questions Suggested | Research Questions to Advance the Use of Photovoice with Children/Youth/ Young Adults with Different Impairments  (6 instances) |  | - Examine how Photovoice can assist nonverbal youth (ASD) in expressing themselves as well as those with more severe intellectual limitations. - ASD occurs along a spectrum, and therefore our findings may not capture youth with ASD with significant verbal communication barriers. Benefits and challenges of using photovoice with youth with ASD may also be useful. - Examine feasibility of using photovoice with students (ASD) with severed language or cognitive impairments. - Examine use of photovoice with young adults with ASD with significant verbal communication barriers. - Given ASD is a spectrum disorder, examine usefulness of photovoice with distinct segments of the continuum and ages within the ASD population - Given ASD is a spectrum disorder, examine usefulness of photovoice with distinct segments of the continuum and ages within the ASD population | (66)  (70)  (81)  (76)  (76)  (76) |
|  |  |  |  |  |
| Future Research Questions Suggested | Research Questions to Build on and Advance the Research Base on NDD Families | The experiences of NDD Children, Youth, and Young Adults in Diverse Settings or Environments  (13 instances) | - Perceived benefits of peer tutoring for youth with ASD overtime. - Explore the secondary education experiences of autistic young people who communicate in other non-verbal ways. - Understand the experience of friendship for adolescents with ASD who have had mainstream schooling and no experience with social skill groups. - Research on facilitators and barriers to after-school physical activity participation should include external measures including collecting data from parents and physical educators. - Examine the characteristics of the physical and community environment that may influence physical activity participation for children with ASD. - Research using objective measures of physical activity levels among youth with CP in rural environments. - There is a need for intervention-based studies to improve physical activity in individuals with CP from rural areas. - Need for evidence-based physical activity interventions at multiple ecological levels. - Examine the role of social support in physical activity participation for individuals with ASD. - This approach [online photovoice] opens avenues for future studies to replicate and explore the lived experi­ences and perspectives of a larger sample size from multiple locations, poten­tially on a nationwide or cross-country scale. - Research on physical activity participation among youth with CP. - Explore the family’s role/familial roles in adolescent development - The well-being of students with ASD. (The well-being of students with ASD remains largely unexplored). | (65)  (80)  (79)  (81)  (81)  (82)  (82)  (82)  (81)  (78)  (82)  (79)  (68) |
| Future Research Questions Suggested | Research Questions to Build on and Advance the Research Base on NDD Families | The Experiences of NDD Children, Youth, and Young Adults (ASD, CP) from Disadvantaged Backgrounds  (11 instances) | - Explore the lives of children with ASD from less privileged backgrounds and in rural communities. - More research is needed to understand the social determinants of health for marginalized populations experiencing health inequities (this was study about Native American young adults with IDD and their caregivers) - To address health inequities, more research is needed to understand health and wellness from the unique perspectives of individuals (young adults) with IDD and those from racial and ethnic minority groups. - Explore the health and wellness experience and perceptions of Native American young adults with IDD (intellectual and/or developmental disabilities) using gender diverse samples. - Explore the health and wellness experience and perceptions of urban dwelling Native American young adults with IDD. - Examine barriers experienced to achieving health and wellness by Native Americans with IDD and their caregivers living in urban communities in achieving health equity. - Find ways of engaging (in research) autistic young people who are harder to reach, especially in times of crisis and emergency. - Future studies on the experience of stress and coping when transitioning to adulthood with diverse samples of youth with ASD (racial, ethnic minorities, various functioning levels) - The implementation of online photovoice in this research offered a significant advantage by enabling the researcher to include geographically dispersed autistic adolescents who would otherwise have been unreachable. This advantage highlights the potential for using online photovoice in future studies with young autistic people. - Additionally, while the participants were diverse in some respects (e.g. school type; family care status), in other respects (e.g. ethnicity) they were not. Future research should seek to work with more diverse samples, but also further explore the way that young people’s experiences vary in relation to demographics, identity and intersectionality. - While this is the first known study to seek the views of adolescents with FASD in the UK, there remains a gap in participatory research across other age groups [this study used online photovoice and focused on youth 12-19yrs]. | (71)  (77)  (77)  (77)  (77)  (77)  (80)  (70)  (78)  (69)  (69) |
| Future Research Questions Suggested | Research Questions to Build on and Advance the Research Base on NDD Families | The Needs of Individuals with ASD and their families  (6 instances) | - Research is needed to acknowledge and support the needs of carers (families of individuals with ASD), including the importance of respite. - Access to competitive employment was important for meaningful participation for individuals with IDD and more research is needed to build work environments that promote long‐term competitive employment. - Explore the need for home modifications for families living with a child with ASD (to build the research base and inform policy and supports). - Explore the home modification needs and experiences of families in private and public housing, and those with limited financial, social, and other forms of supports. - Examine the impact of home adaptations on siblings and children with ASD themselves - Qualitative research of individuals’ views of transition into early adulthood would contribute to an understanding of what could improve experiences and outcomes for young people’s education, independent living, mental health, and employment, and concurrently reduce risks for young people and adults with FASD. | (74)  (77)  (74)  (74)  (74)  (69) |
| Future Research Questions Suggested | Research Questions to Build on and Advance the Research Base on NDD Families | The Experience of Assistance Dog Ownership for Individuals with ASD and their Families  (5 instances) | - Explore the best way of supporting families to prepare for having an assistance dog and adjust to this significant change. - Identify the benefits of canines on physical activity in individuals with ASD. - Identifying challenges of assistance dog ownership. - Large experimental studies are required to determine the effectiveness of assistance dogs on improving family quality of life and outcomes for children on the autism spectrum. - Future studies should explore the use of animals to promote social engagement for children and youth with ASD in the clinical setting | (72)  (81)  (72)  (72)  (67) |
| Future Research Questions Suggested | Research Questions to Build on and Advance the Research Base on NDD Families | Engage NDD Families in Program and Research Development  (3 instances) | - Future research [using online photovoice] could incorporate measures for participants to self-evaluate their roles in a research process. - Incorporating more opportunities for families, children and adults with CP and other mobility impairments to develop specific programing is crucial to ensure accessibility of programs, equipment, and public spaces. - Inviting Native Americans with IDD and their caregivers to share their perspectives offers insight to facets that influence their engagement with activities that promote positive health and wellness. | (69)  (82)  (77) |
| Future Research Questions Suggested | No Future Questions Noted |  | - None noted | (73, 80) |
